# Supplementary material for: MoS2 nanopore identifies single amino acids with sub-1 Dalton resolution
Source: Nat Commun. 2023 May 20;14:2895. doi: 10.1038/s41467-023-38627-x (PMC10199900; doi:10.1038/s41467-023-38627-x)
Supplement: Supplementary file 1 — Supplementary Information [file 41467_2023_38627_MOESM1_ESM.pdf]

Supplementary Information

for

MoS_2 Nanopore Identifies Single Amino Acids with Sub-1 Dalton Resolution

Fushi Wang[1], Chunxiao Zhao[1], Pinlong Zhao[1], Fanfan Chen[1], Dan Qiao[1], Jiandong Feng[1,2]*

[1]*Laboratory of Experimental Physical Biology, Department of Chemistry, Zhejiang University, 310027 Hangzhou, China*

[2]*Research Center for Quantum Sensing, Research Institute of Intelligent Sensing, Zhejiang Lab, 311121 Hangzhou, China*

**Correspondence should be addressed to jiandong.feng@zju.edu.cn*

Table of contents

Supplementary notes 4

1. Stability and validity of experimental system 4
2. Pore size and region of sensitivity 5
3. Nanopore heterogeneity 8
4. Single Amino Acids Identification Network (SAAINet) 10

Supplementary figures 13

1. A summary of the experimental results for twenty amino acids 13
2. Mean relative current blockade versus molecular volumes in a same MoS_2 nanopore. 14
3. Schematic illustration of the region of sensitivity 15
4. Current traces for amino acid translocation recorded under different voltages 16
5. The influence of voltage on the translocation of amino acids 17
6. Characterizations of single-layer MoS_2 18
7. Optical images of MoS_2 transferred to SiN_x substrates 19
8. I-V characteristics of MoS_2 nanopore Devices #1-15 20
9. I-V characteristics of MoS_2 nanopore Devices #16-30 21
10. Stability of MoS_2 nanopores 22
11. Identification of F, Y and W in differently sized pores 23
12. Current noise power spectra for a ~0.5 nm diameter MoS_2 nanopore 24
13. A comparison of current noise power spectra under different voltages for a ~1.58 nm diameter MoS_2 nanopore 25
14. Typical current traces of amino acids translocating through a MoS_2 nanopore 26
15. An overlap of 100 waveforms of the translocation for G and A 27
16. The architecture of SAAINet 28
17. Illustration of the model input 29
18. Over sampling method 30
19. Classification performance of different inputs 31
20. Classification performance of the different ML algorithms 32
21. Schematics for electrophoretically driving the amino acids 33
22. Reproduced experiments of the electrically charged amino acids 34
23. Schematics of the different entering orientation of amino acids in MoS_2 nanopores 35
24. Reproduced experiments of the hydrophobic non-aromatic amino acids 36

25. Reproduced experiments of polar uncharged amino acids..37
26. Reproduced experiments of hydrophobic aromatic amino acids 38
27. Reproduced experiments of G and P..39
28. Dwell time of translocations for of L and I..40
29. Dwell time of translocation for L, I, M, F, Y and W 41
30. Discrimination of mixed T and Y in a MoS_2 nanopore 42
31. Reproduced experiments of D and N, E and Q, N and L...43
32. Representative fragments of current traces for the translocation of Y and p-Y.............44
33. For the same amino acids in the same device, the results of $\Delta I/I_0$ do not change over time 45
Supplementary tables...46
34. The hyper-parameter settings of SAAINet network 46
Supplementary references 47

1. Stability and validity of experimental system

We fabricated more than 150 MoS_2 nanopore devices. To ensure the reliability of translocation data, the experiments under different voltages were carried out. From the current traces recorded at 200 mV, 250 mV and 300 mV, we found that amplitudes of current blockades increased as the voltage increased (Device #2, Supplementary Fig. 4), and when the voltage is 300 mV, the interval between events decreased obviously. As shown in the scatter plot in Supplementary Fig. 5b, c, ΔI increased with the increase of voltage from 150 mV to 200 mV, while $\Delta I/I_0$ remained the same (Device #16). Besides, we calculated the frequency of translocation events of S at 50, 100, 150, 200 mV (Device #5, Supplementary Fig. 5a), and the results show that with the increase of external potential, the frequency of translocation events increased and gradually tended to be saturated. Due to the higher noise caused by higher potential (Device #5, Supplementary Fig. 13), we found that voltages in the range of 150-200 mV are suitable for the amino acid translocation experiments.

In order to eliminate the influence of measurement order for detection, the experiment of changing measurement order (A-B-A experiment) was carried out. We performed the experiment on Y first, and then on F, finally on Y again in Device #25 to show the robustness of single amino acid identification. In this group of experiments, though the size of nanopore increased to a certain extent over time, the experimental finding remained unchanged, that is, the relative current blockade caused by Y was always larger than that caused by F (Supplementary Fig. 26a, b).

For the MoS_2 nanopores with a long-term stability, the histograms of $\Delta I/I_0$ for the same amino acids do change over time (Device #10, Supplementary Fig. 33).

2. Pore size and region of sensitivity

The control of the appropriate size for nanopores is critical to the sensitivity of the nanopore. As most of our nanopores are made using the ECR method[1, 2], to provide information of the pore size, we displayed the current-voltage (I-V) characteristics of all MoS_2 nanopore devices that are mentioned in this article in Supplementary Figs. 8, 9. The sizes of nanopores were estimated based on the I-V data using the empirical nanopore conductance formula[3, 4]. For sub-nanometer pores, the I-V characteristics can exhibit a nonlinear behavior which is consistent with the earlier report[5]. MoS_2 nanopore devices we fabricated featured a long-term stability. We counted the number of events measured in a typical device within 48 hours and the measured I-V characteristics before and after the experiments showed that the nanopore did not considerably change in size (Device #15, Supplementary Fig. 10).

For the MoS_2 nanopore devices used to read out all the twenty amino acids, the diameter of nanopores should be larger than 0.7 nm considering that several amino acids have aromatic rings in their structures. For the identification of amino acids with small volumes, such as G and A, a relatively small nanopore ~0.5 nm is used (Device #4, Fig. 1f, Supplementary Fig. 15). When the size of the nanopore is close to that of the amino acids to be measured, it is conducive to distinguish the samples. The nanopores used in the single amino acid identification experiments range from sub-nanometer to 1.6 nm. If the size of a nanopore is larger than 1.6 nm, distinguishing different amino acids from the histograms of relative current blockades becomes challenging due to the lack of sufficient resolution. In Supplementary Fig. 11, we show that the identification

capability of the nanopore is related to the pore size. When the pore size is ~1.58 nm, the relative current blockade of F, Y, W increased with the increase of molecular volume (Device #5, Supplementary Fig. 11a). When the diameter of nanopore is ~1.80 nm, the distribution of $\Delta I/I_0$ for F and Y overlapped, but W could still be identified from the three sets of data (Device #17, Supplementary Fig. 11b). When the size of nanopore is larger than 2 nm, Y and W cannot be distinguished (Device #18, Supplementary Fig. 11c).

We found that the current blockades induced by glycine (G), Gly-Gly (GG) and Gly-Gly-Gly (GGG) were almost the same (Device #3, Fig.1d). As sketched in Supplementary Fig. 3, for a nanopore, when the region of sensitivity is equal to the size of a single amino acid, the exclude volumes for homopeptides of different lengths are close. If the region of sensitivity for a nanopore is larger than a single amino acid, homopeptides of different lengths show length-dependent current blockades, as in aerolysin nanopores[6, 7].

In fact, whether the sensing region is only single amino acids depends on the size of MoS_2 nanopore. The diameter of MoS_2 nanopore largely determines the sensitivity. Chen et al.[8] observed the stepwise translocation process of peptides in a 2.2 nm MoS_2 nanopore with molecular dynamics (MD) simulations and the step length is 3 amino acid residues. Si et al.[9] observed the stepwise translocation process of peptides in a 1.4 nm MoS_2 nanopore with steered molecular dynamics (SMD) simulations and each amino acid residue induces only one step of current. With the decrease of pore size, the region of sensitivity in MoS_2 nanopore becomes smaller, and thus the spatial resolution

can be improved. In the experiment we mentioned in Fig.1d and Supplementary Fig. 3, the pore size is smaller than 1.4 nm, and thus it is theoretically possible to achieve the resolution close to one amino acid.

A computational study by Aluru et al.[10] has considered a similar MoS_2 nanopore system as in our experimental work, and has made several conclusions that agree with our experiment. For example, as the diameter of nanopore increases, the sensitivity decreases significantly, and higher volume (or mass) of amino acids mostly leads to larger current blockade. Their MD simulations results show that MoS_2 nanopore has the potential to detect individual amino acids in a polypeptide chain.

3. Nanopore heterogeneity

Considering the heterogeneity of MoS_2 nanopore devices, reproduced measurements across different MoS_2 nanopore devices were performed for each groups of amino acids. Our results show that the order of $\Delta I/I_0$ caused by each type of amino acids remains consistent in different devices. The reproduced data are shown in Supplementary Figs. 22, 24-27.

K and R with pI of 9.74 and 10.76 are the only positively charged amino acids in the buffer of pH 7.8. R always shows a larger relative current blockade than K (Device #6, Supplementary Fig. 22a), which is consistent with their molecular volumes. For D, E and H, the $\Delta I/I_0$ of D and E is close, and H could easily be distinguished in all the devices (Devices #5, 20, 21, Supplementary Fig. 22b-d). We also noticed that for the electrically charged amino acids the dual peaks can be observed frequently, which may be caused by the two charged groups in their chains, leading to two preferred translocation orientations[11] (Supplementary Fig. 23).

The reproduced experiments on hydrophobic non-aromatic amino acids are shown in Supplementary Fig. 24. This group of amino acids contains structural isomers, L and I, the results for which may related to the size of nanopores. From the histograms of $\Delta I/I_0$ for A and L, V and L (Devices #24, 18, Supplementary Fig. 24c, d), the relative current blockades are positively correlated with the volumes of these amino acids.

For the group of polar uncharged amino acids, the difference between S and T is one $-CH_3$ group, and between Q and N is one $-CH_2-$ group. We have mentioned in the main text that the MoS_2 nanopores have a high sensitivity to chemical groups, and in

Supplementary Fig. 25c, we show the histograms of $\Delta I/I_0$ for these four amino acids, and the relative current blockades are positively correlated with the volumes of the molecules (Device #10). Note that for T and N, their molecular volumes are very close, 118.3×10^{-3} nm^3 and 120.1×10^{-3} nm^3, respectively. This result demonstrates that our nanopore resolution can reach ~2×10^{-3} nm^3, and the average identification accuracy of T and N reaches 82.69% (Supplementary Fig. 25d).

The reproduced experiments on the hydrophobic aromatic amino acids and the special group of amino acids show consistent results (Devices #5, 25-27, Supplementary Figs. 11, 26, 27).

Dwell time of translocation is another important characteristic often used in nanopore sensing of biomolecules. The event dwell time histograms were fitted by an exponential decay function (Devices #5, 8, 17, 22, Supplementary Figs. 28, 29). The reason we did not use dwell time as the main criteria is that the dwell time for amino acid translocation is too short, mostly shorter than 2 ms. In addition, dwell time difference can be caused by a number of effects (including charge, chemical properties, size, etc.) and it is not clear to serve as a robust identification criterion for differentiation.

Note that the histograms of $\Delta I/I_0$ of G and A in 3 out 10 devices show obvious bimodal distribution (Devices #12, 24, Fig. 2h, Supplementary Fig. 24c), and these two have the smallest volumes of all twenty amino acids. Our preliminary conjecture is that hydrogen bonds may cause the formation of glycine-water or alanine-water complex[12, 13] which introduces the bimodal distribution.

4. Single Amino Acids Identification Network (SAAINet)

Deep neural network architecture. SAAINet takes the raw ionic current data and five features obtained by Transalyzer[14] as input. We concatenate both the ionic current and the features to form the input representation:

$$\mathbf{X} = [\mathbf{x_1}, \mathbf{x_2}, \cdots, \mathbf{x_N}] \tag{1}$$

$$\mathbf{x_n} = [i_n, f_1, f_2, f_3, f_4, f_5]^T \tag{2}$$

where N is the length of an event, and i_n is the n-th current value in the event. The LSTM is employed to model the sequence characteristic of the ionic current. After feeding the input representation to LSTM, the hidden state $\mathbf{h_t}$ of LSTM are represented as:

$$\mathbf{i_t} = \sigma(\mathbf{W_i^x} \cdot \mathbf{x_t} + \mathbf{W_t^h} \cdot \mathbf{h_{t-1}} + \mathbf{b_i}) \tag{3}$$

$$\mathbf{f_t} = \sigma(\mathbf{W_f^x} \cdot \mathbf{x_t} + \mathbf{W_f^h} \cdot \mathbf{h_{t-1}} + \mathbf{b_f}) \tag{4}$$

$$\mathbf{o_t} = \sigma(\mathbf{W_o^x} \cdot \mathbf{x_t} + \mathbf{W_o^h} \cdot \mathbf{h_{t-1}} + \mathbf{b_o}) \tag{5}$$

$$\mathbf{c_t} = \mathbf{f_t} \odot \mathbf{c_{t-1}} + \mathbf{i_t} \odot \tanh(\mathbf{W_c^x} \cdot \mathbf{x_t} + \mathbf{W_c^h} \cdot \mathbf{h_{t-1}} + \mathbf{b_c}) \tag{6}$$

$$\mathbf{h_t} = \mathbf{o_t} \odot \tanh(\mathbf{c_t}) \tag{7}$$

where $\mathbf{W_i^x}$, $\mathbf{W_t^h}$, $\mathbf{W_f^x}$, $\mathbf{W_f^h}$, $\mathbf{W_o^x}$, $\mathbf{W_o^h}$, $\mathbf{W_c^x}$, $\mathbf{W_c^h} \in R^{d\times 1}$ are the weight matrices of LSTM, and $\mathbf{b_i}$, $\mathbf{b_f}$, $\mathbf{b_o}$, $\mathbf{b_c} \in R^{d\times 1}$ are the biases of LSTM. $\mathbf{i_t}$, $\mathbf{f_t}$, $\mathbf{o_t}$, and $\mathbf{c_t}$ denote the input, forget and output gates respectively. σ represents the sigmoid function, and $\odot$ is the element-wise multiplication. By LSTM, the hidden vectors [$\mathbf{h_1}$, $\mathbf{h_2}$, …, $\mathbf{h_N}$] are obtained as the representation of current signals. Then, the initial whole representation of an event is obtained by average pooling of the hidden states:

$$\bar{\mathbf{h}} = \sum_{t=1}^{N} \mathbf{h_t} \tag{8}$$

To improve the fitting capability of the neural network and allow high level feature inference, four fully connected layer are utilized on the global event representation $\bar{h}$.

$$\mathbf{f^1} = \text{ReLU}(\mathbf{W_1} \cdot \bar{\mathbf{h}} + \mathbf{b_1}) \tag{9}$$

$$\mathbf{f^2} = \text{ReLU}(\mathbf{W_2} \cdot \mathbf{f^1} + \mathbf{b_2}) \tag{10}$$

$$\mathbf{f^3} = \text{ReLU}(\mathbf{W_3} \cdot \mathbf{f^2} + \mathbf{b_3}) \tag{11}$$

$$\mathbf{f^4} = \text{ReLU}(\mathbf{W_4} \cdot \mathbf{f^3} + \mathbf{b_4}) \tag{12}$$

where $\mathbf{W_1} \in R^{d_{f1} \times d}$, $\mathbf{W_2} \in R^{d_{f2} \times d_{f1}}$, $\mathbf{W_3} \in R^{d_{f3} \times d_{f2}}$, $\mathbf{W_3} \in R^{d_c \times d_{f3}}$ are the weight matrix, and $\mathbf{b_1} \in R^{d_{f1}}$, $\mathbf{W_2} \in R^{d_{f2}}$, $\mathbf{W_3} \in R^{d_{cf3}}$, $\mathbf{W_3} \in R^{d_c}$ are the bias to be learned during training. The ReLU is an activation function which performs ReLU(x) = max(0, x). Afterwards, the predicted probability of the j-th amino acid category $j \in [1, C]$ is computed by a softmax function:

$$\mathbf{y_i'} = \frac{\exp(\mathbf{f_i^4})}{\sum_{i=1}^{C} \mathbf{f_i^4}} \tag{13}$$

SAAINet is trained in an end-to-end way in a supervised learning framework. During training, all the parameters are optimized to minimize the loss function. Let y is the true labels of events regarding the categories of amino acids. The through entropy is use to define the loss function, and the formula is as follows:

$$\text{loss} = \sum_{i=1}^{C} y_i \log(y_i') \tag{14}$$

where y_i is a one-hot label of the i-th class, and $y_i \in [0,1]$ is the predicted probability of i-th class.

Data sets and experimental settings. The model input is descripted in Supplementary Fig. 17. Besides, the over sampling method is used to balance the data, which is illustrated in Supplementary Fig. 18. In order to achieve better model

performance, the suitable hyper-parameters are summarized through experiments, which are listed in Supplementary Table. 1.

Results on different inputs and methods. Supplementary Fig. 19 shows the performance comparison of these models with different inputs. It can be observed that the model using all the four parts, i.e. I, $F_{1,2}$, $F_{3,4,5}$ and $E100$, as inputs achieves the best performance. Comparing I, $I+F_{1,2}$, $I+F_{1,2}+F_{3,4,5}$ and $I+F_{1,2}+F_{3,4,5}+E100$, the performance of the models is improved, which indicates that all the four input parts are important to achieve an effective model.

Supplementary Fig. 20 presents the main results between our proposed method and other four methods. From the result, we can see, comparing with the other two traditional methods, RF has the best results in general. Moreover, for a certain input, SAAINet is always better than the other four methods.

Groups	Amino acids		Molecular mass (g/mol)	Molecular volume ($*10^{-3}$ nm^3)	Results
Electrically charged amino acids	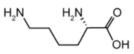	Lys (**K**)	146.19	172.7	$\mathbf{K}_{<\Delta I/I_0>} < \mathbf{R}_{<\Delta I/I_0>}$
	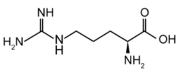	Arg (**R**)	174.20	188.2	
	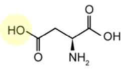	Asp (**D**)	133.11	115.4	
	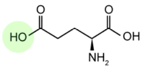	Glu (**E**)	147.13	140.9	$\mathbf{D}_{<\Delta I/I_0>} \approx \mathbf{E}_{<\Delta I/I_0>} < \mathbf{H}_{<\Delta I/I_0>}$
	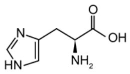	His (**H**)	155.15	156.3	
Hydrophobic non-aromatic amino acids	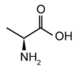	Ala (**A**)	89.09	87.8	
	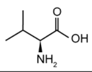	Val (**V**)	117.15	138.8	$\mathbf{A}_{<\Delta I/I_0>} < \mathbf{V}_{<\Delta I/I_0>} < \mathbf{L}_{<\Delta I/I_0>}$
	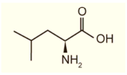	Leu (**L**)	131.18	168.0	
	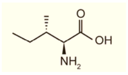	Ile (**I**)	131.18	166.1	$\mathbf{L}_{<\Delta I/I_0>} < \mathbf{I}_{<\Delta I/I_0>} < \mathbf{M}_{<\Delta I/I_0>}$
	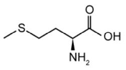	Met (**M**)	149.21	165.2	In 2 out of 7 devices, $\mathbf{L}_{<\Delta I/I_0>} > \mathbf{I}_{<\Delta I/I_0>}$
Polar uncharged amino acids	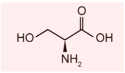	Ser (**S**)	105.09	91.7	
	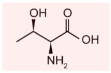	Thr (**T**)	119.10	118.3	$\mathbf{S}_{<\Delta I/I_0>} < \mathbf{T}_{<\Delta I/I_0>} < \mathbf{N}_{<\Delta I/I_0>} < \mathbf{Q}_{<\Delta I/I_0>}$
	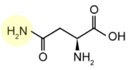	Asn (**N**)	132.12	120.1	
	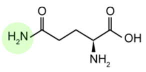	Gln (**Q**)	146.15	145.1	
Hydrophobic aromatic amino acids	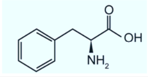	Phe (**F**)	165.20	189.7	
	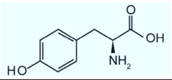	Tyr (**Y**)	181.19	191.2	$\mathbf{F}_{<\Delta I/I_0>} < \mathbf{Y}_{<\Delta I/I_0>} < \mathbf{W}_{<\Delta I/I_0>}$
	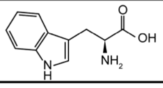	Trp (**W**)	204.20	227.9	
Other amino acids	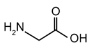	Gly (**G**)	75.07	59.9	
	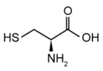	Cys (**C**)	121.16	105.4	$\mathbf{G}_{<\Delta I/I_0>} < \mathbf{C}_{<\Delta I/I_0>} < \mathbf{P}_{<\Delta I/I_0>}$
	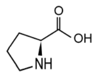	Pro (**P**)	115.13	123.3	

Supplementary Fig. 1. A summary of the experimental results for all twenty amino acids. The data of molecular volume is from ref [15].

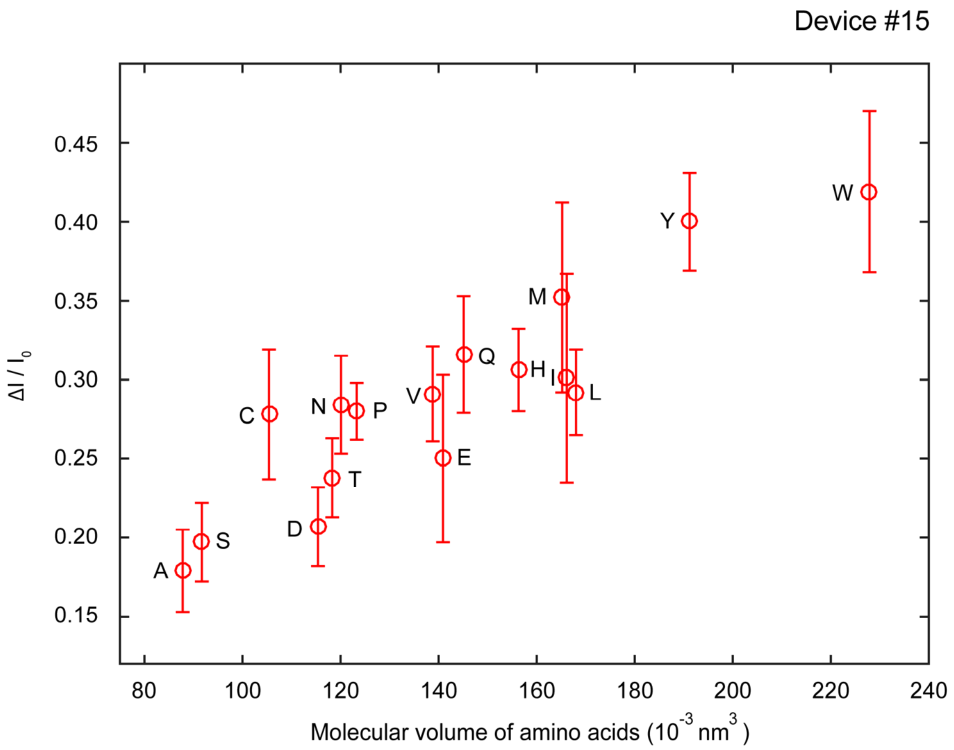

Supplementary Fig. 2. Mean relative current blockade versus molecular volume in a same MoS_2 nanopore (Device #15).

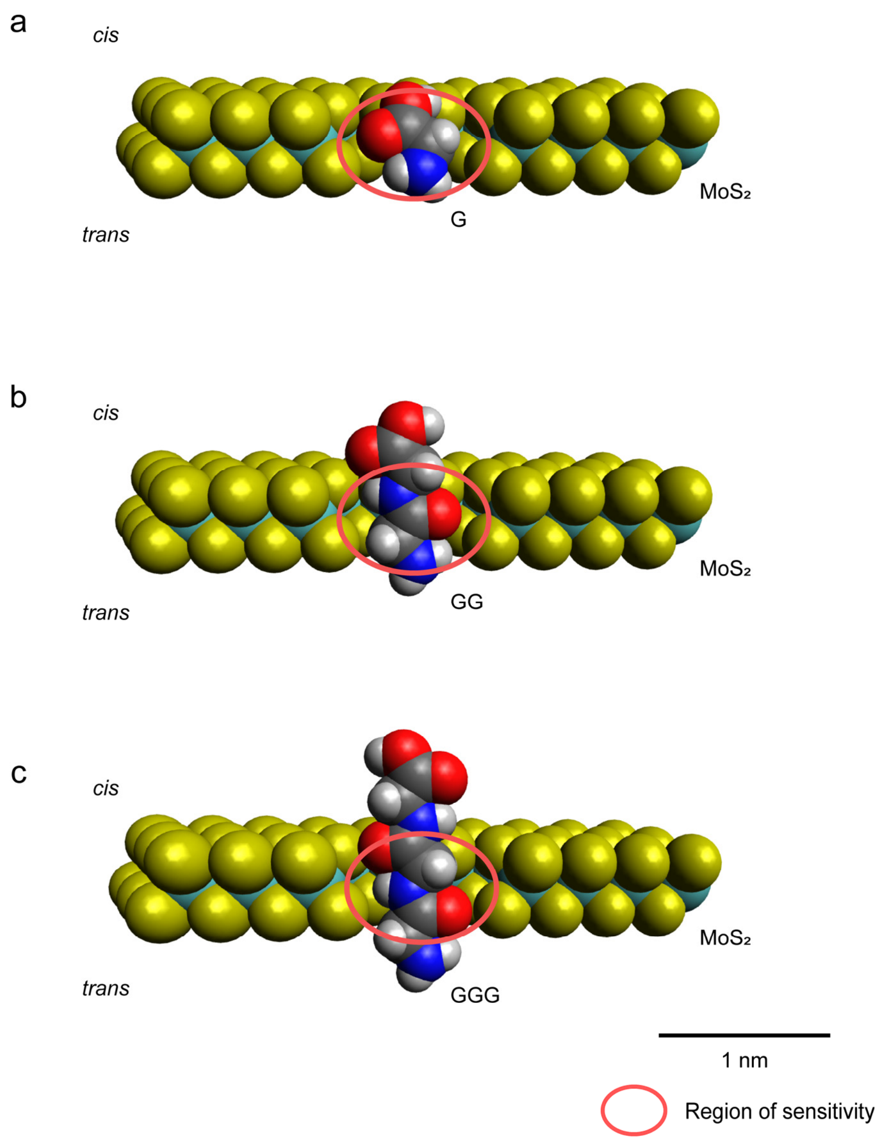

Supplementary Fig. 3. Schematic illustration of the region of sensitivity of single-layer MoS_2 nanopores. The translocations of G **(a)**, GG **(b)** and GGG **(c)** in single-layer MoS_2 nanopores.

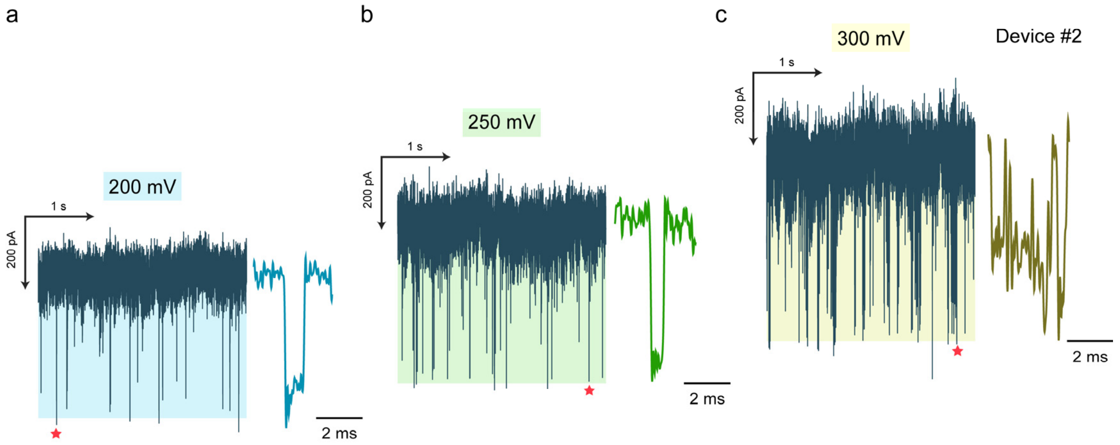

Supplementary Fig. 4. Representative current traces for amino acid translocation recorded under different voltages. Traces of translocation of A in Device #2 recorded under **(a)** 200 mV, **(b)** 250 mV, **(c)** 300 mV.

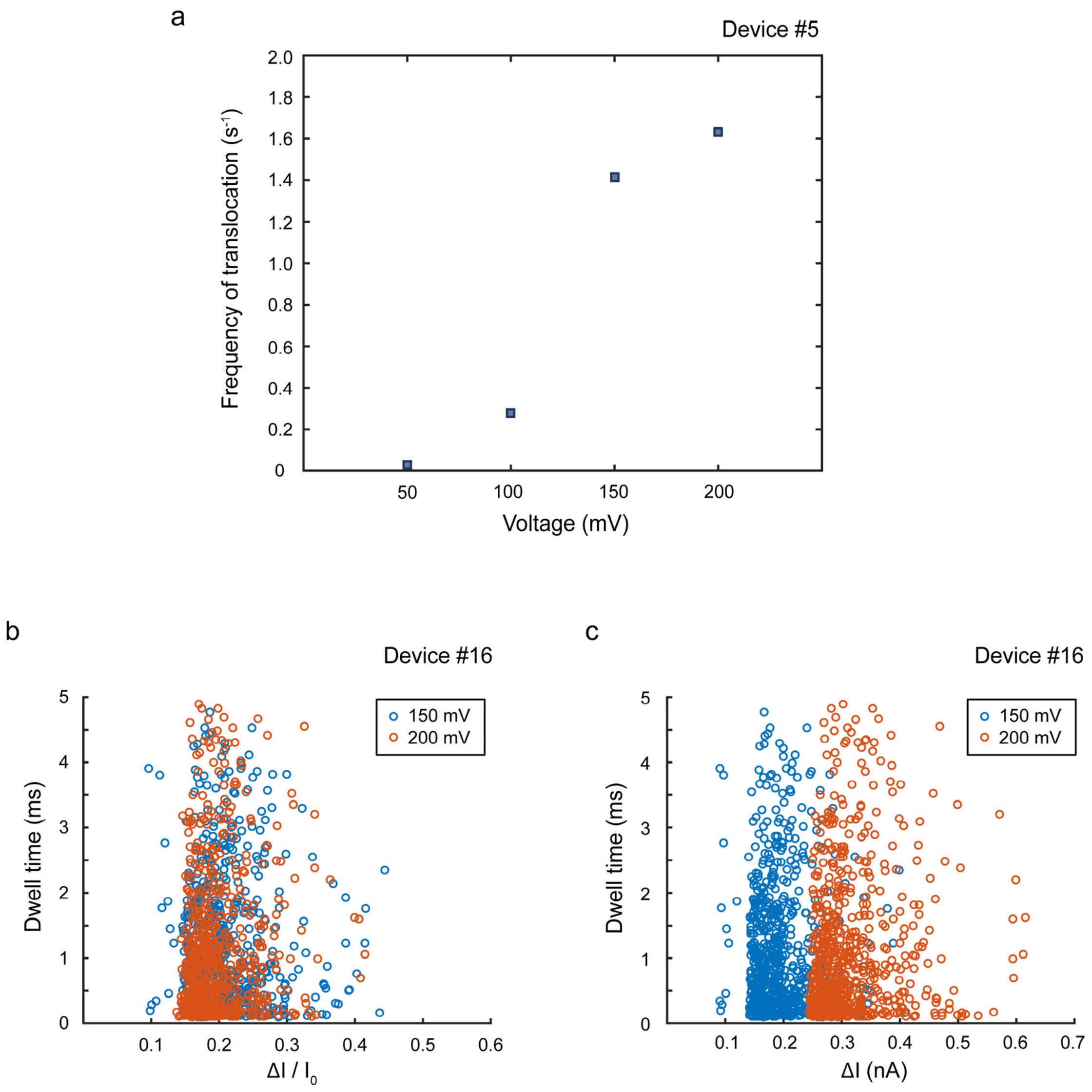

Supplementary Fig. 5. The influence of voltage on the translocation of amino acids. (a) The frequencies of translocation for S in Device #5 under 50, 100, 150, 200 mV, respectively. **(b-c)** The current blockades increase with the increasing of voltages for the translocation of A, while $\Delta I/I_0$ remains the same (Device #16).

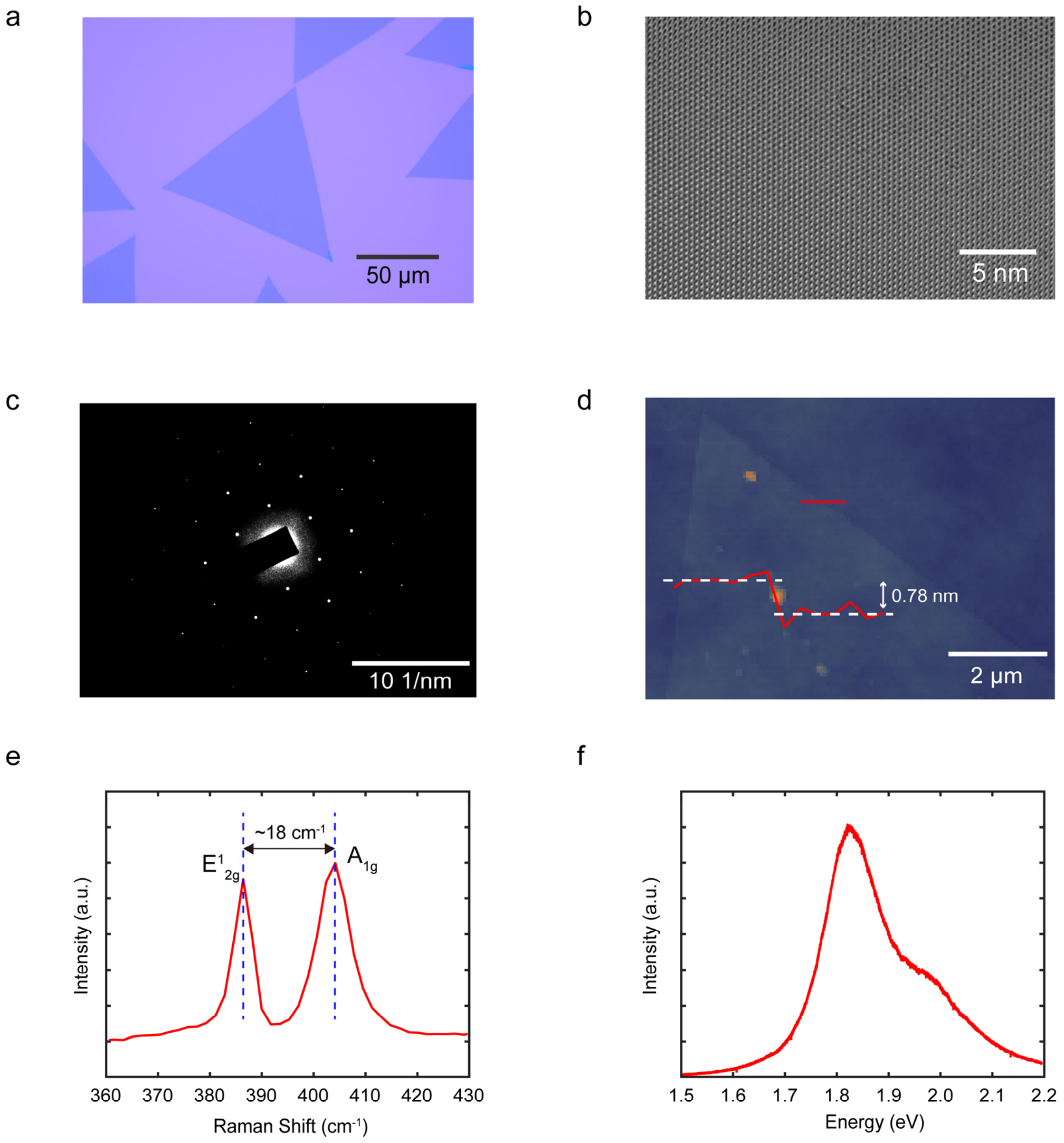

Supplementary Fig. 6. Characterizations of single-layer MoS_2. (a) Optical images of single-layer MoS_2 grown on SiO_2/Si by CVD. **(b)** High resolution spherical aberration corrected transmission electron microscope (AC-TEM) image of a monolayer MoS_2 membrane. **(c)** Electron diffraction pattern of single crystal monolayer MoS_2 membrane acquired by TEM operating at 200 kV. **(d)** Atomic force microscopy image of MoS_2 sample transferred on sapphire. Magnified red trace shows the thickness profile along the red line, and the height is about 0.78 nm. **(e)** Raman spectrum of monolayer MoS_2 grown on SiO_2/Si. The two peaks reveal the Raman shift for out-of-plane vibration of S atoms (A_{1g}) at ~404 cm^{-1} and in-plane vibration of Mo and S atoms (E^1_{2g}) at ~386 cm^{-1}. **(f)** Photoluminescence spectrum of monolayer MoS_2 grown on SiO_2/Si. The excitation wavelength is 532 nm. The observed frequency difference (~18 cm^{-1}) in Raman spectrum and the position of peak (~1.82 eV) in photoluminescence spectrum shows that MoS_2 sample is monolayer with high optical quality and uniformity.

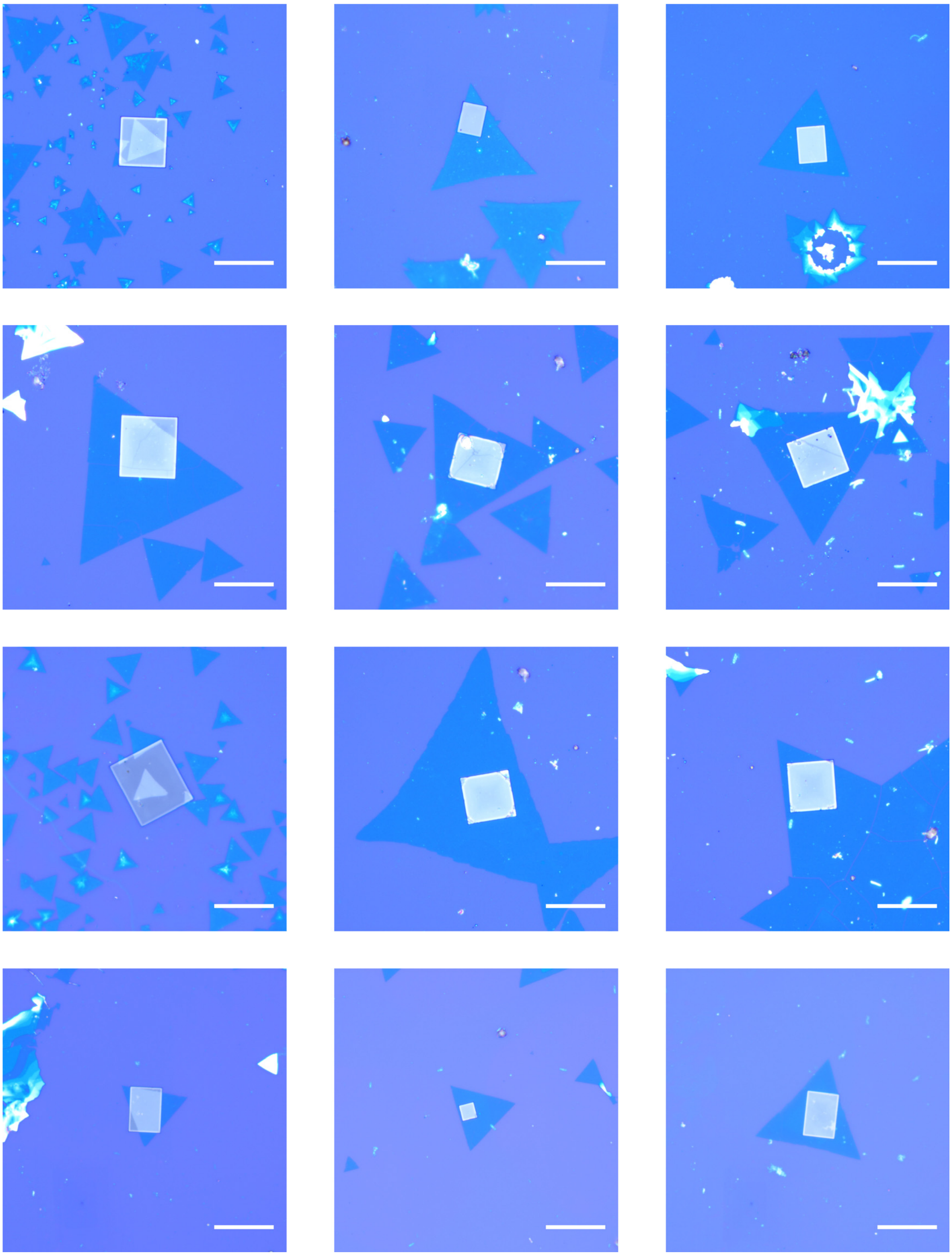

Supplementary Fig. 7. Optical images of MoS_2 transferred to SiN_x substrates. Scale bar: 20 μm.

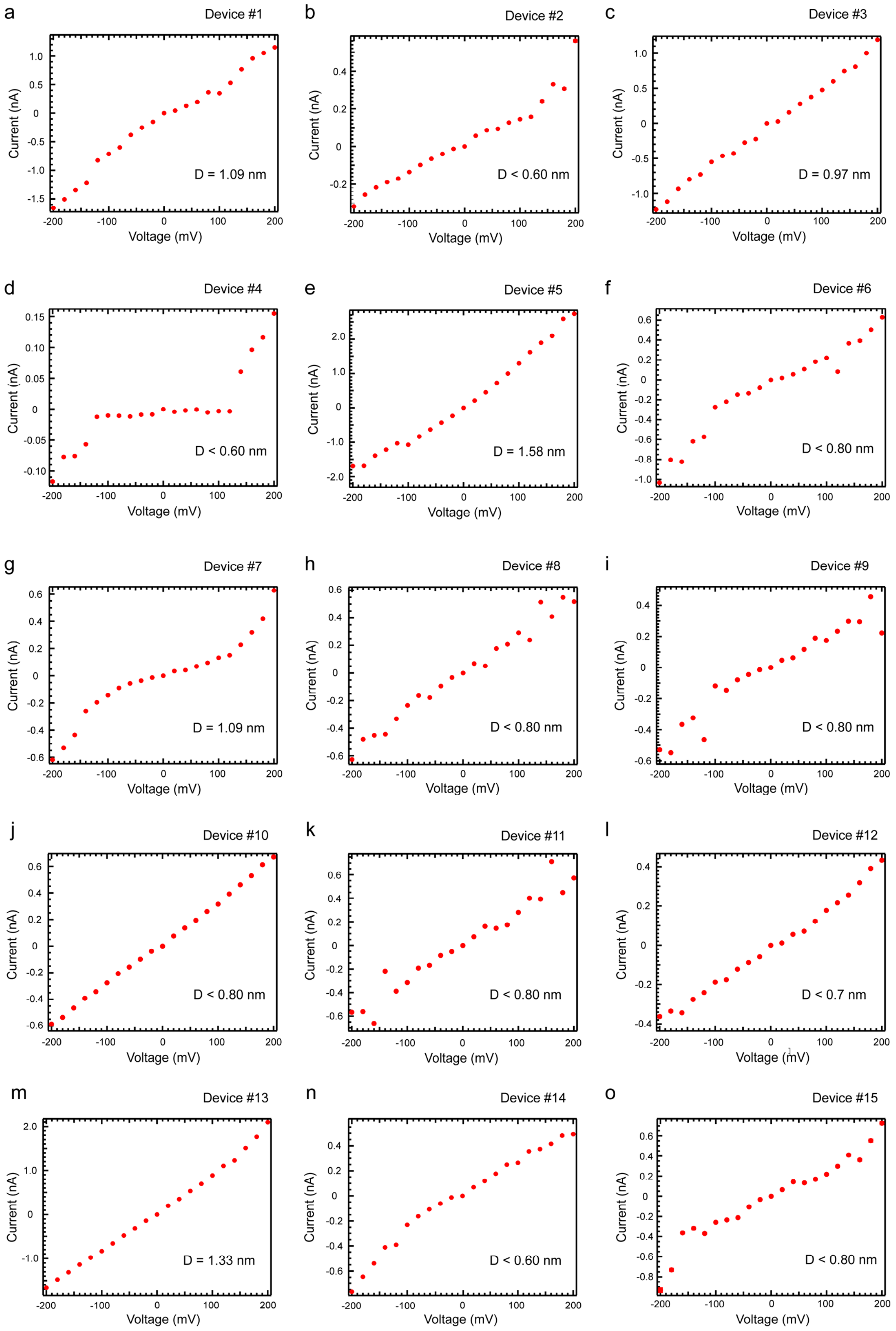

Supplementary Fig. 8. I-V characteristics of MoS_2 nanopore Devices #1-15 (panels a-o respectively).

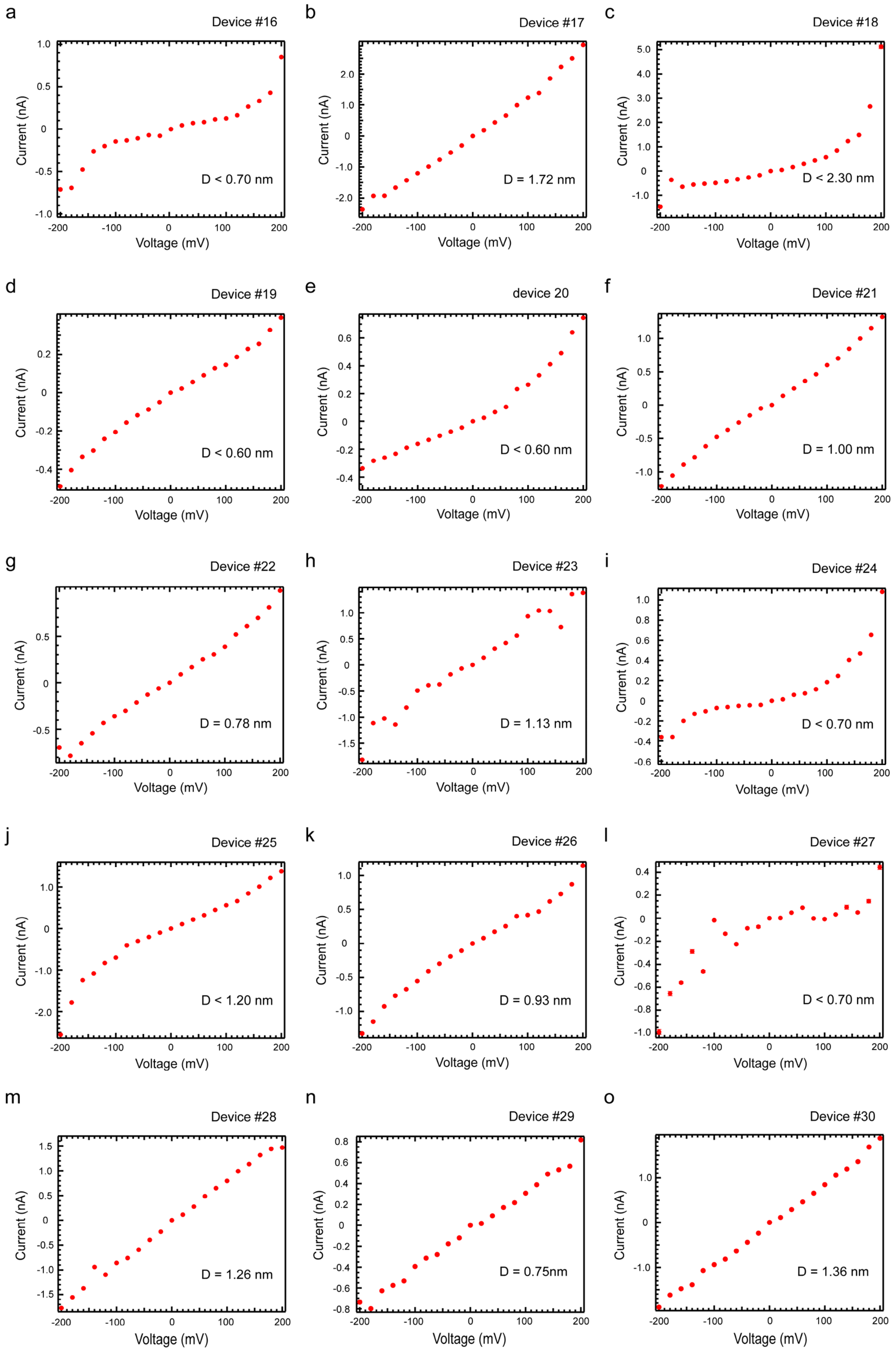

Supplementary Fig. 9. I-V characteristics of MoS_2 nanopore Devices #16-30 (panels a-o respectively).

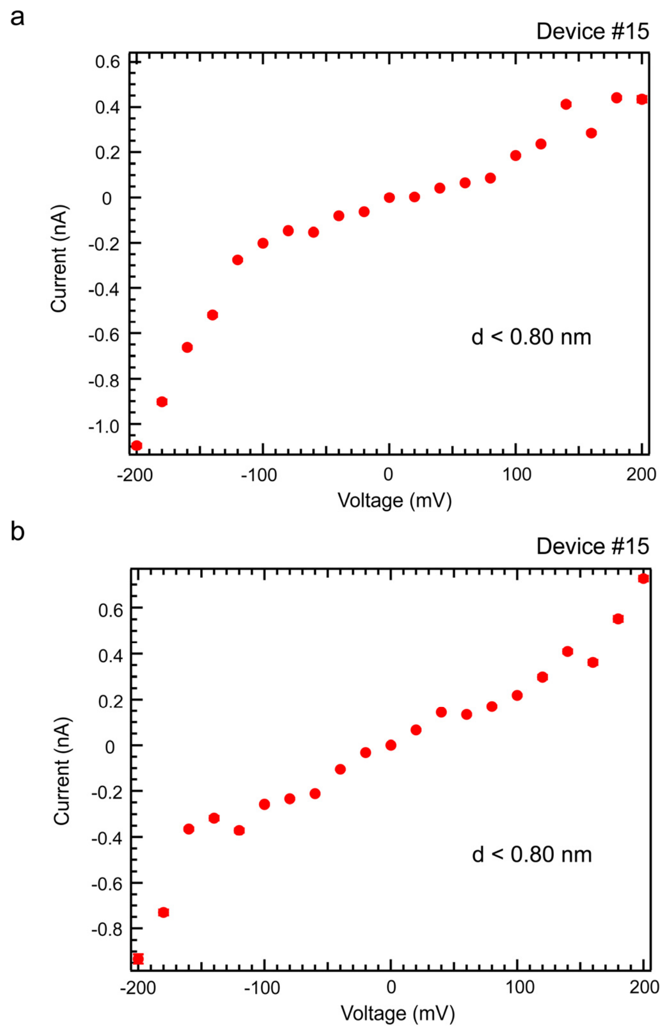

Supplementary Fig. 10. Stability of a typical MoS_2 nanopore. I-V characteristics measured before **(a)** and after **(b)** the translocation experiments of twenty amino acids in Device #15.

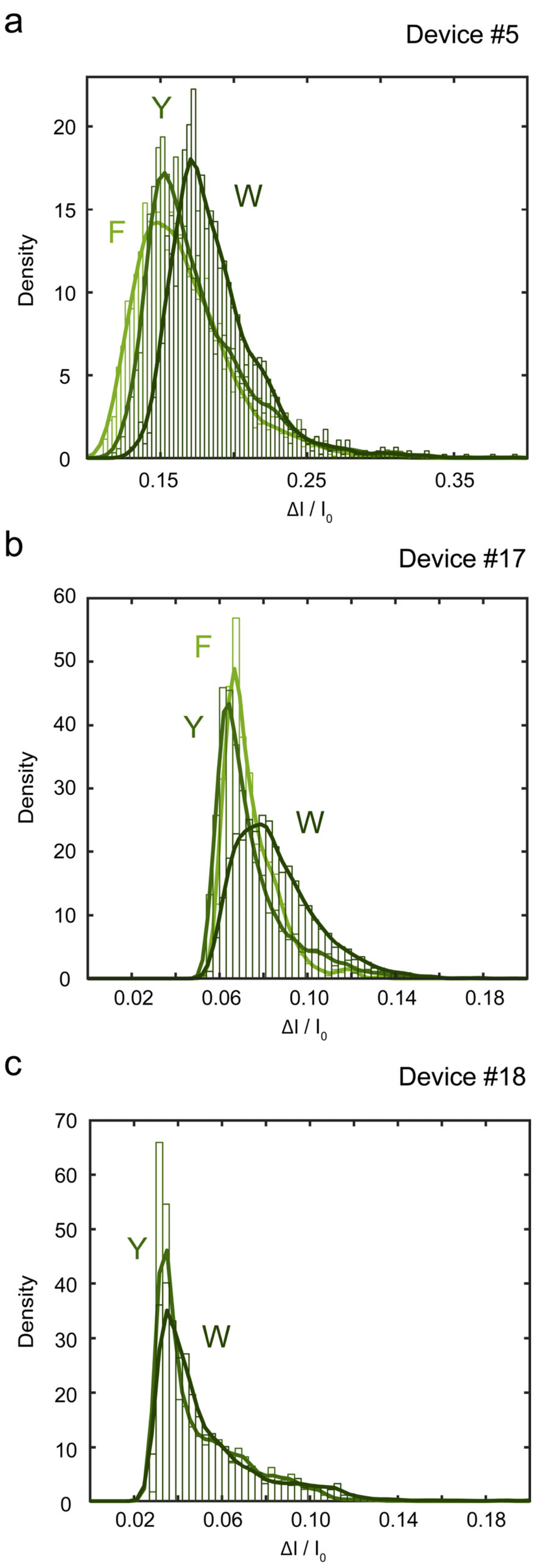

Supplementary Fig. 11. Identification of F, Y and W in differently sized pores. (a) The diameter of Device #5 is ~1.58 nm, **(b)** The diameter of Device #17 is ~1.86 nm, **(c)** The diameter of Device #18 is ~2.10 nm.

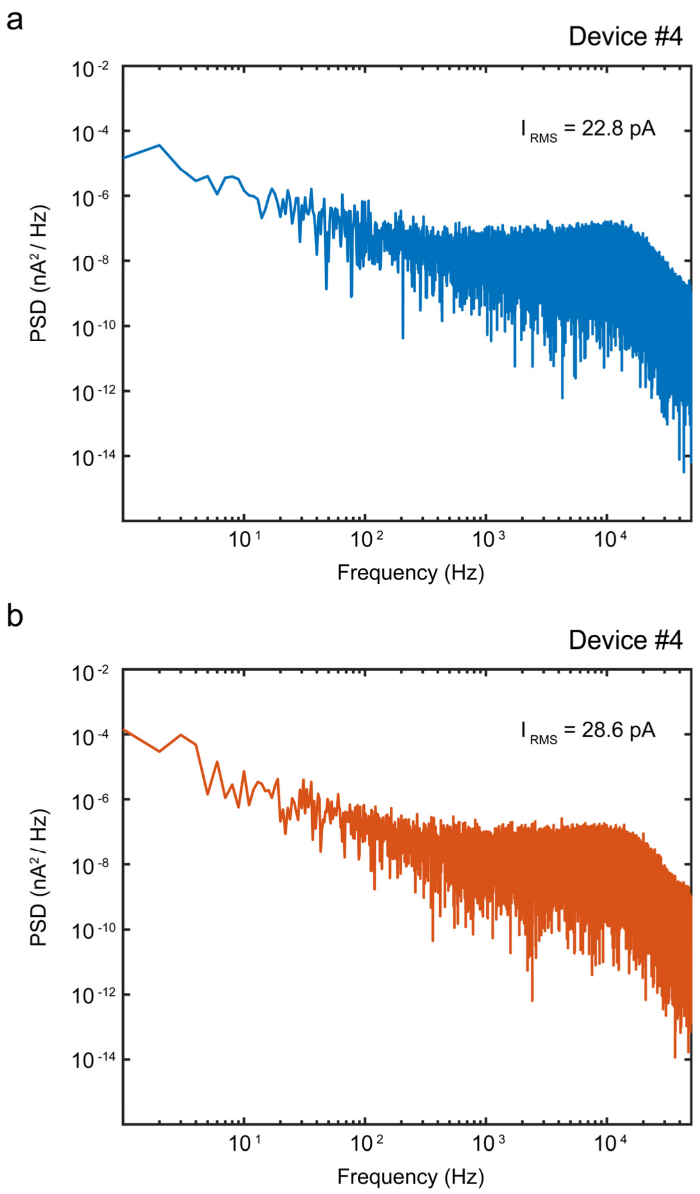

Supplementary Fig. 12. Current noise power spectra for a ~0.5 nm diameter MoS_2 nanopore. (a) In the buffer solution without amino acids at 200 mV (Device #4), **(b)** When signals occurred after amino acids (A) added at 200 mV (Device #4).

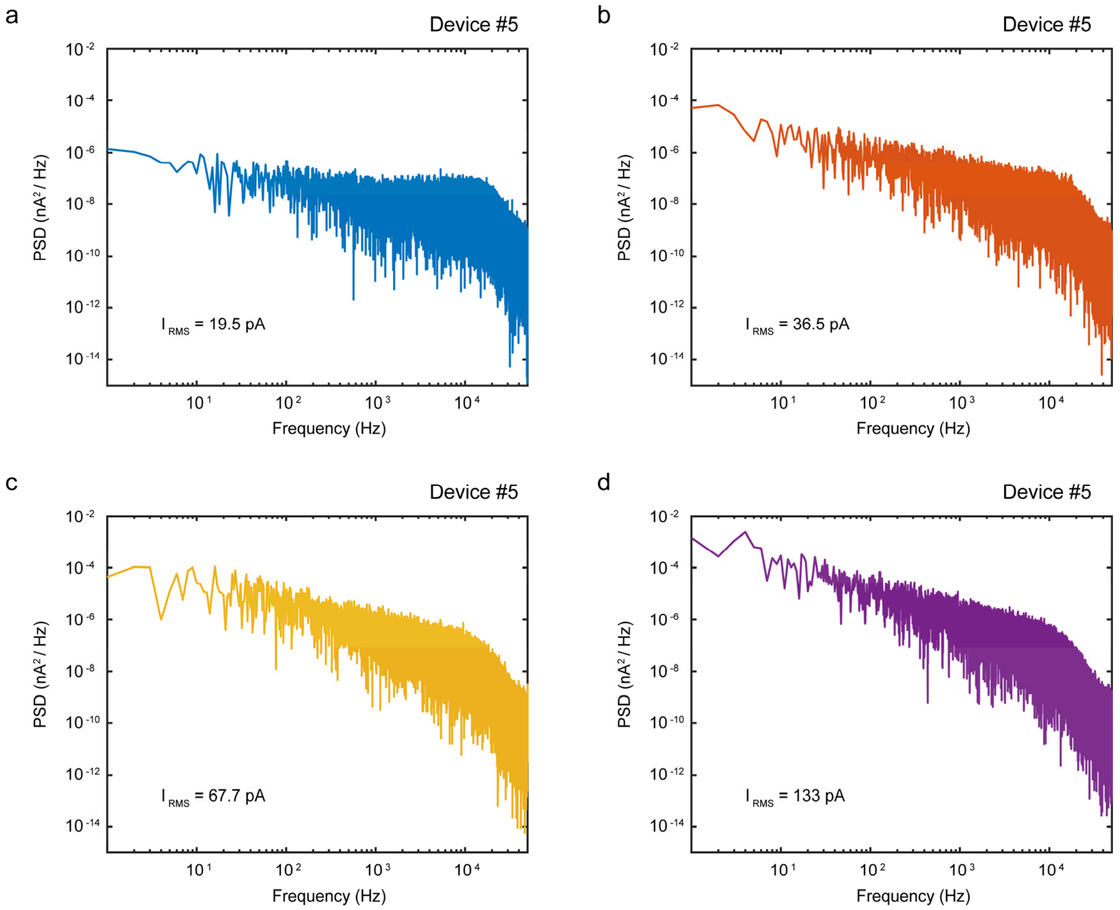

Supplementary Fig. 13. A comparison of current noise power spectra under different voltages for a ~1.58 nm MoS_2 nanopore (Device #5). (a) 20 mV, **(b)** 80 mV, **(c)** 140 mV and **(d)** 200 mV. During translocation, a larger noise was caused by higher potential, and thus the external potential should not be too high.

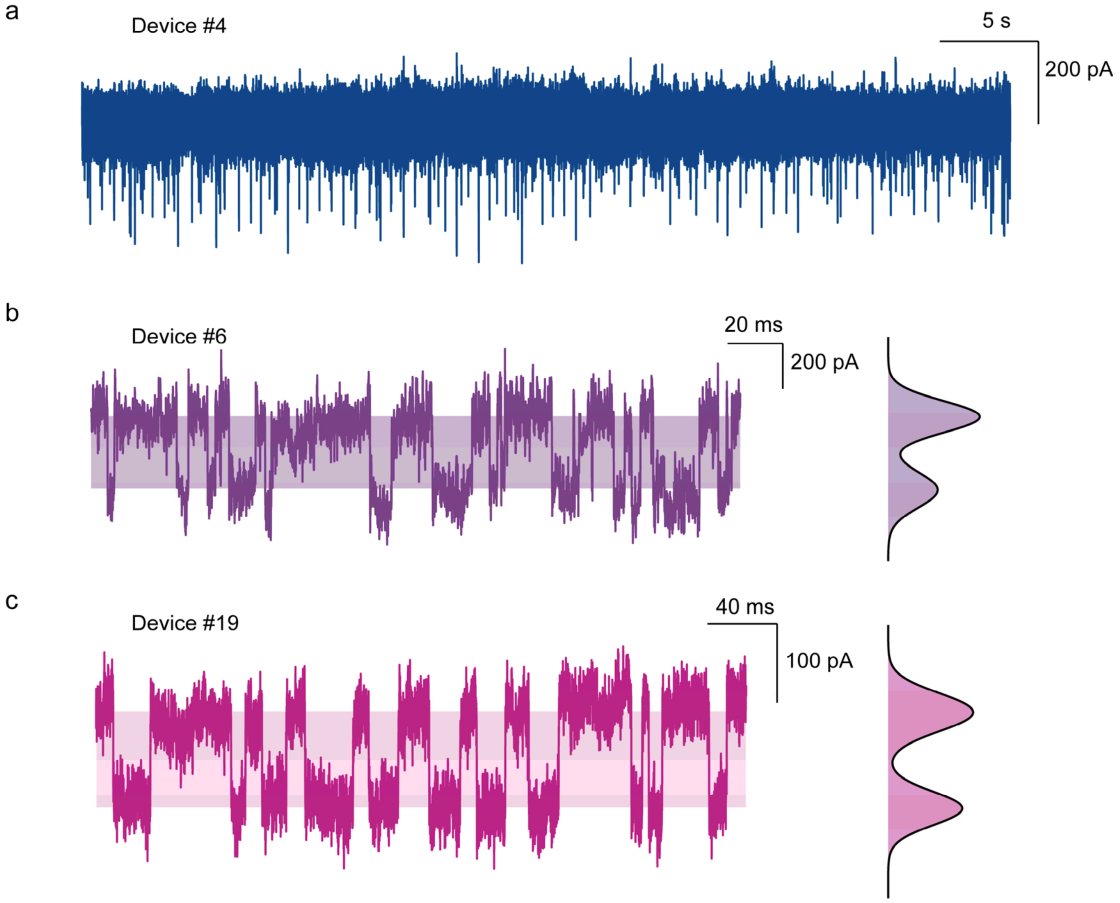

Supplementary Fig. 14. Typical current traces of amino acids translocating through a MoS_2 nanopore. (a) Ion current trace of the translocation of S (Device #4). **(b)** and **(c)** show the similar trace to Fig. 1c, this phenomenon usually occurs when the diameter of nanopore is close to the size of detected amino acids. For **(b)**, pore size is ~0.65 nm (Device #6), and the amino acids detected are F. For **(c)**, pore size is ~0.5 nm (Device #19), and the amino acids detected are G. Besides, **(c)** is recorded under 300 mV, which means this phenomenon could be observed under different voltages.

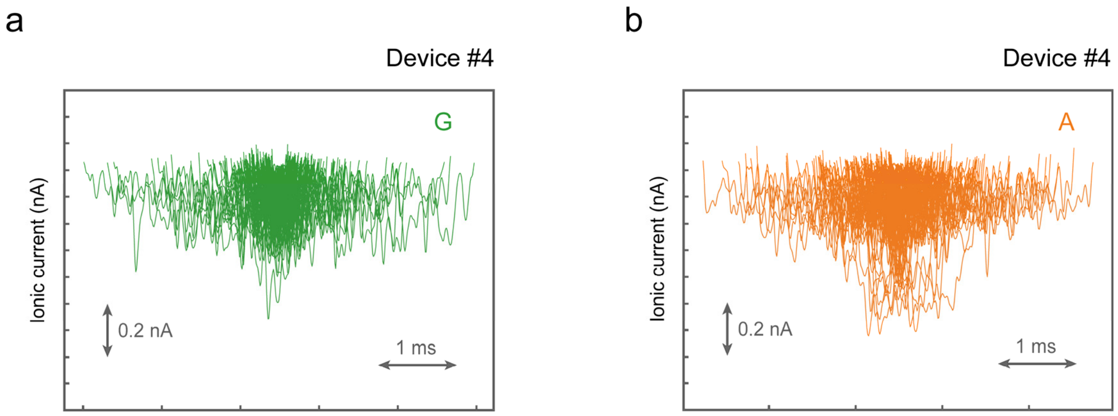

Supplementary Fig. 15. An overlap of 100 waveforms of the translocation for G (a) and A (b) in Device #4.

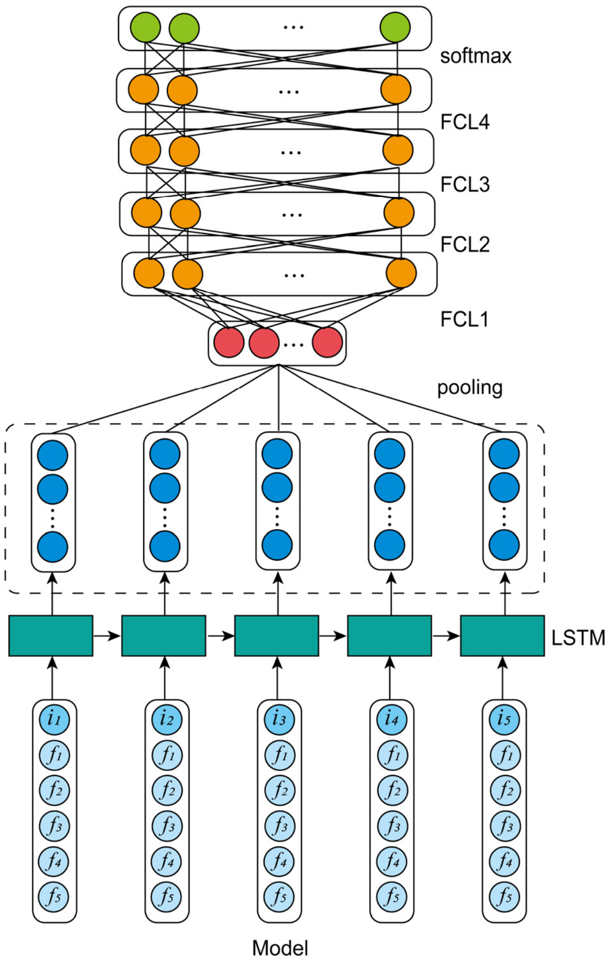

Supplementary Fig. 16. The architecture of SAAINet. SAAINet consists of the input layer, the long short-term memory (LSTM), the pooling layer, the four fully connected layers (FCL) and the softmax layer as output.

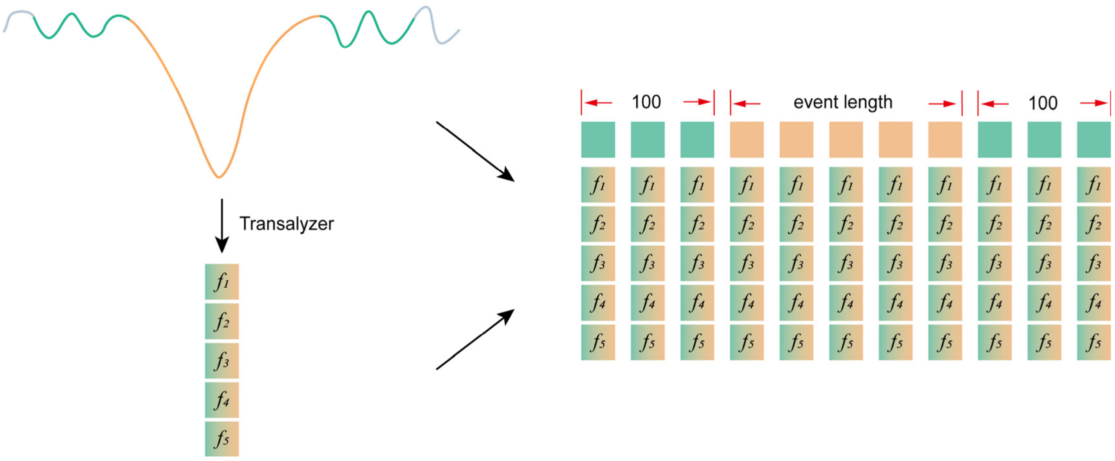

Supplementary Fig. 17. Illustration of the model input. The five features are obtained from Transalyzer[14], a Matlab GUI-based package. f_1 is the relative current blockade. f_2 is the event dwell time. f_3, f_4, f_5 are the average current blockade, the integrated area (integral) of event and the normalized baseline, respectively. In order to obtain additional useful information, the model also accounts multiple current values before and after the event as input.

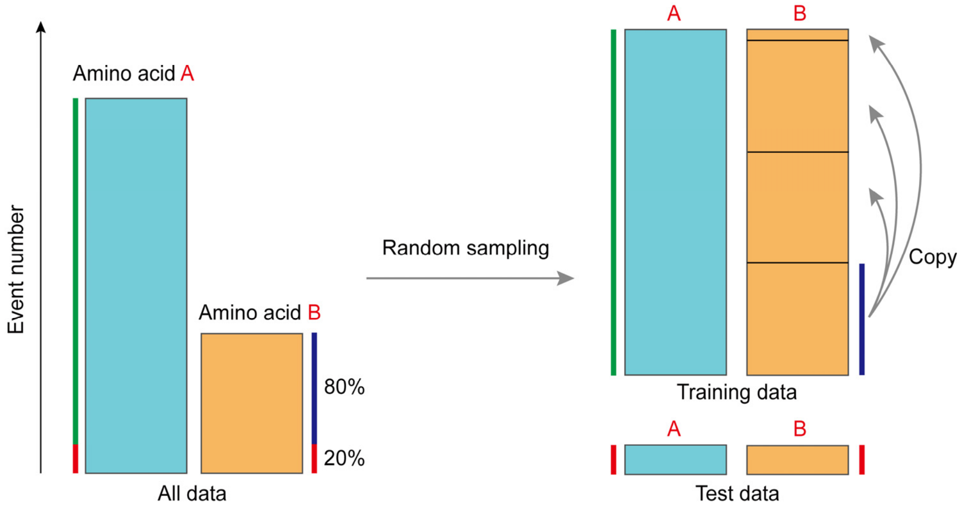

Supplementary Fig. 18. Over sampling method. To eliminate the bias of unbalanced data when training a machine learning model, the method replicates the samples from minority classes to balance the data.

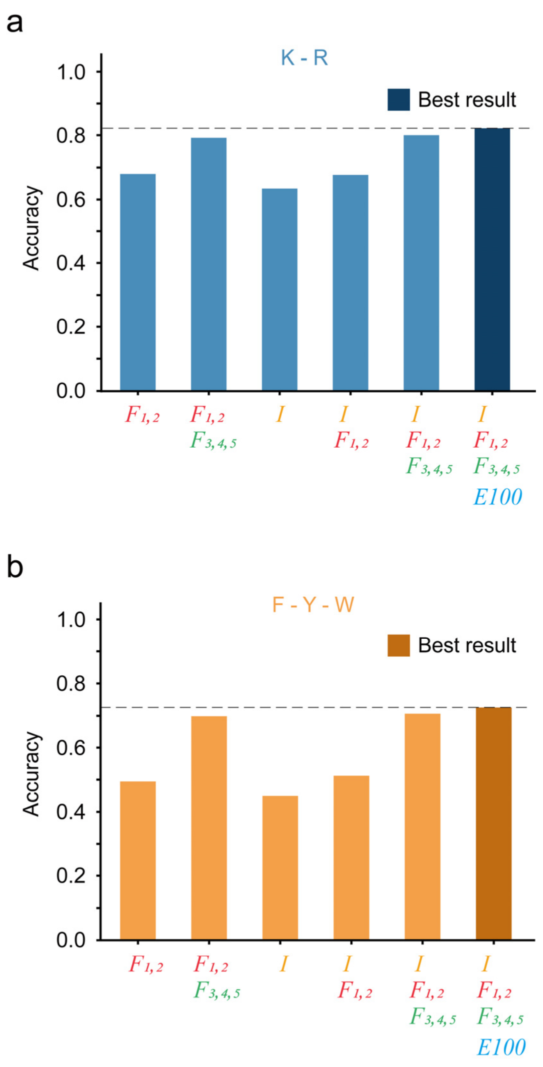

Supplementary Fig. 19. Classification performance of different inputs. **(a)** Performance of different inputs for the classification of K and R. **(b)** Performance of different inputs for the classification of F, Y and W. $F_{n,m}$ means both f_n and f_m are used. *E100* denotes extra 100 points of current trace before and after each event.

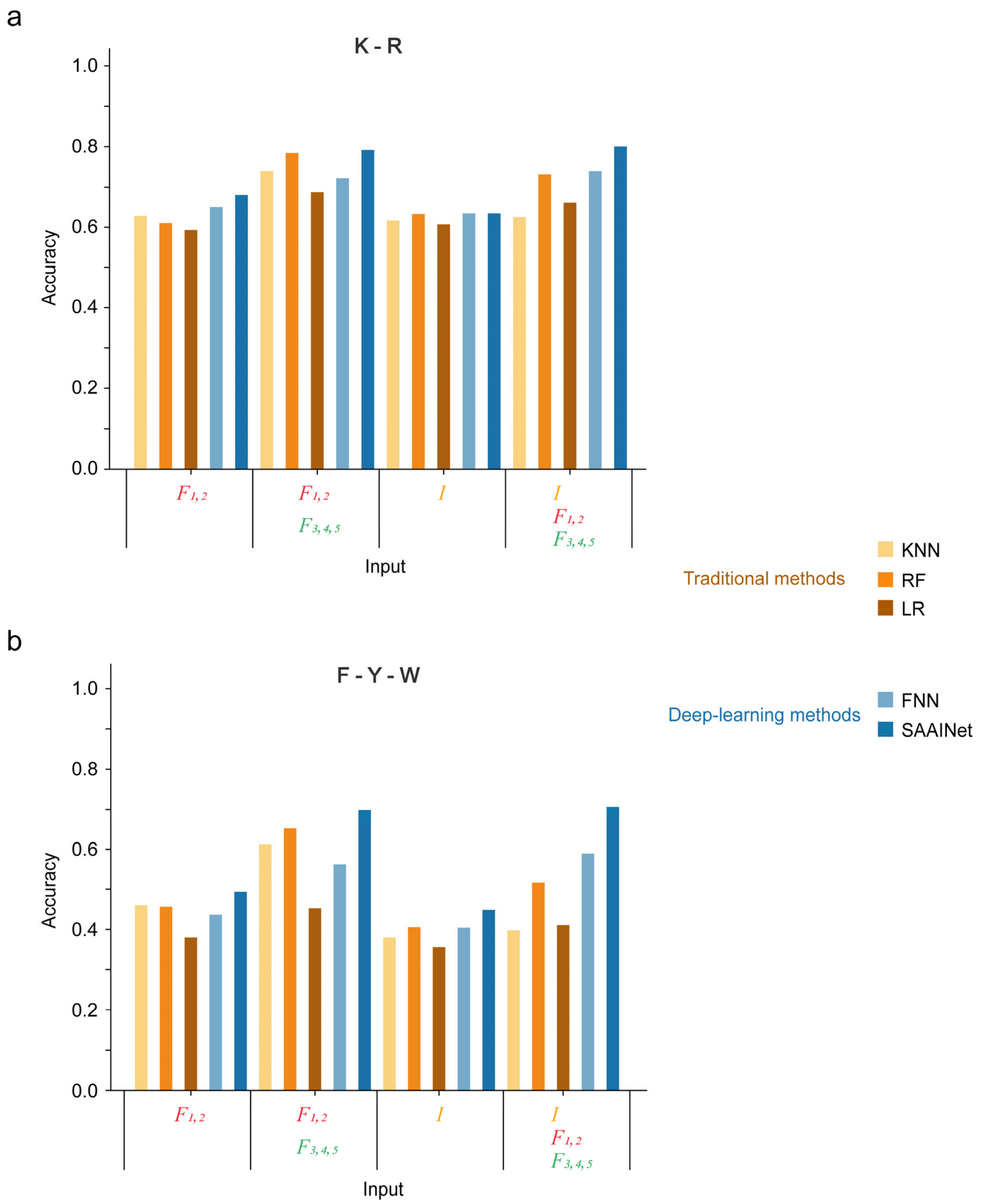

Supplementary Fig. 20. Classification performance of the different ML algorithms. (a) Performance of the different ML algorithms for the classification of K and R. **(b)** Performance of the different ML algorithms for the classification of F, Y and W. K-nearest neighbor (KNN), random forest (RF) and logistic regression (LR) belong to traditional methods. Fully connected neural network (FNN) and our proposed network (SAAINet) are deep-learning methods.

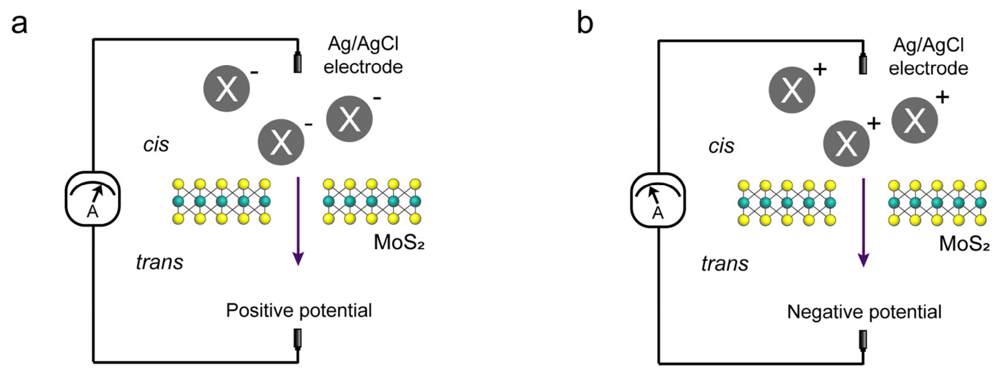

Supplementary Fig. 21. Schematics for electrophoretically driving the amino acids carrying opposite charges in the buffer solution with pH 7.8. The X in **(a)** represents amino acids with pI < 7.8: D, E, H, A, V, I, L, M, S, T, N, Q, F, Y, W, G, C, P. The X in **(b)** represents amino acids with pI > 7.8: K and R. The results of K and R were acquired under -200 mV applied to the trans compartment due to their positive charges at pH 7.8, and that of the remaining 18 amino acids were obtained under +200 mV due to their negative charges at pH 7.8.

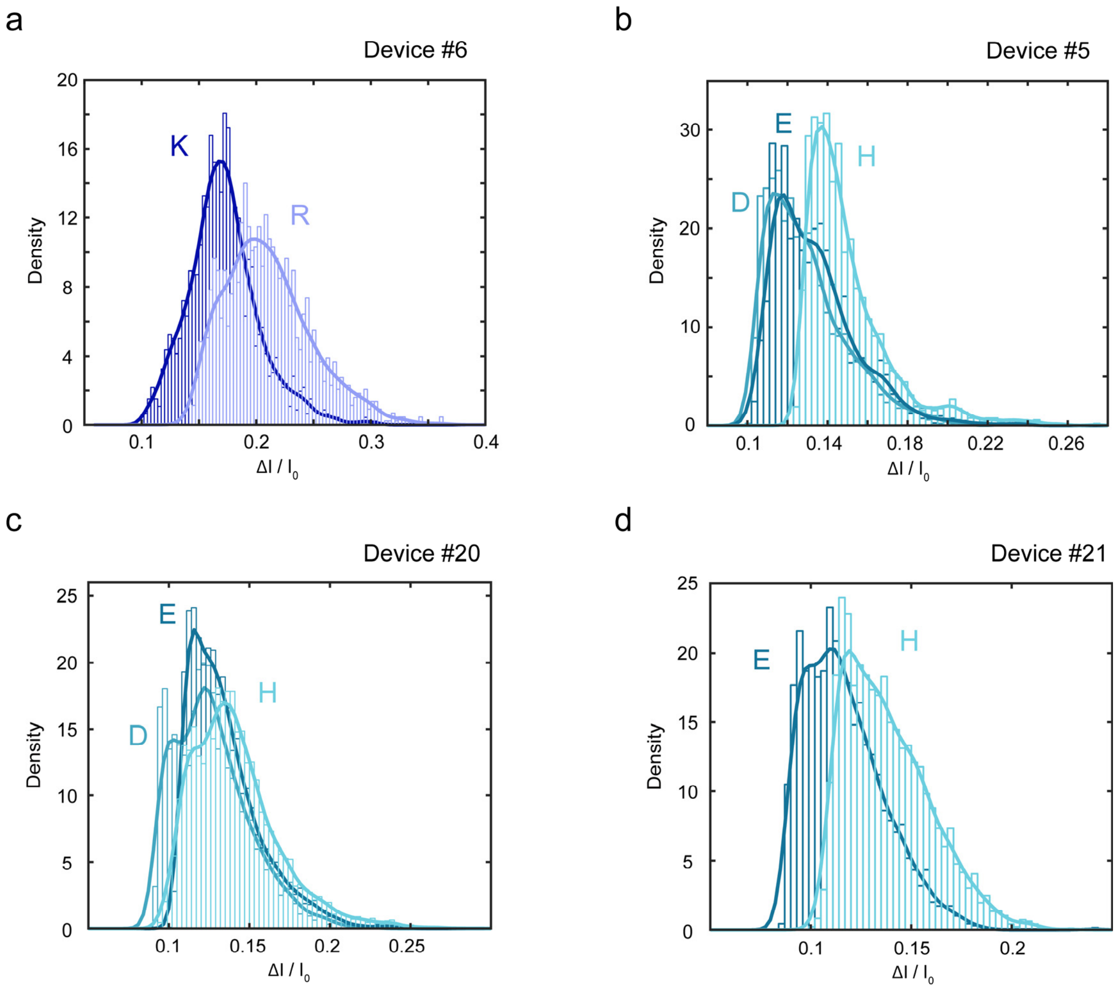

Supplementary Fig. 22. Representative reproduced experiments of the electrically charged amino acids. (a) Reproduced experiments of K and R in Device #6. **(b-d)** Reproduced experiments of D, E and H in Device #5, #20 and #21.

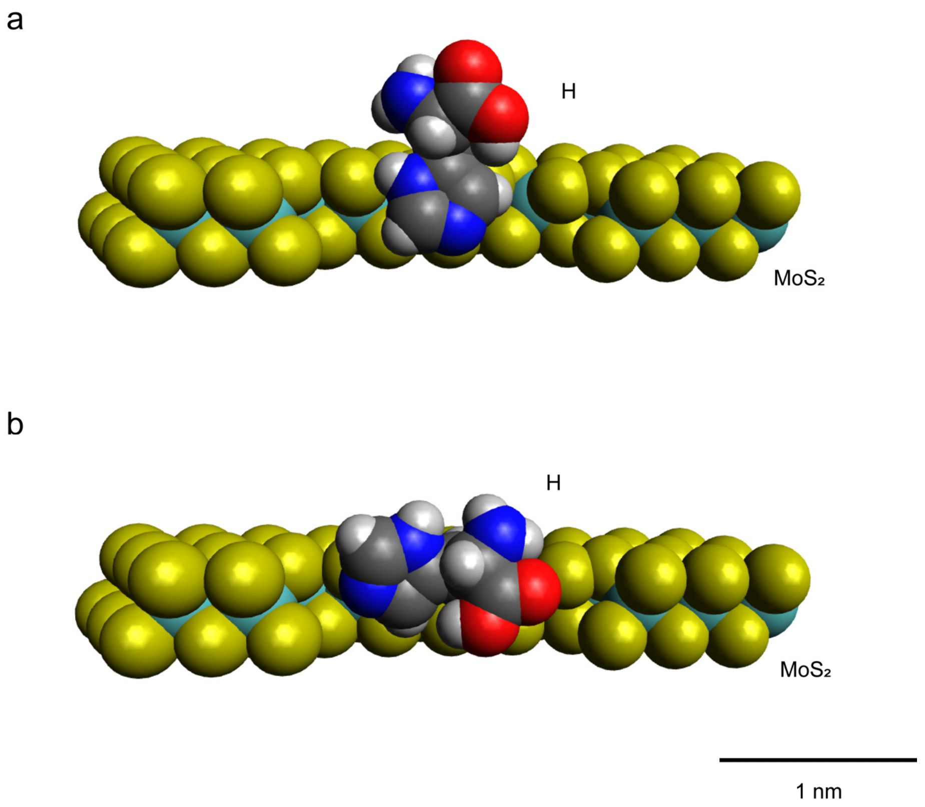

Supplementary Fig. 23. Schematics of the different entering orientations of amino acids in MoS_2 nanopores. (a-b) Side views of H in a MoS_2 nanopore illustrating the two different orientations.

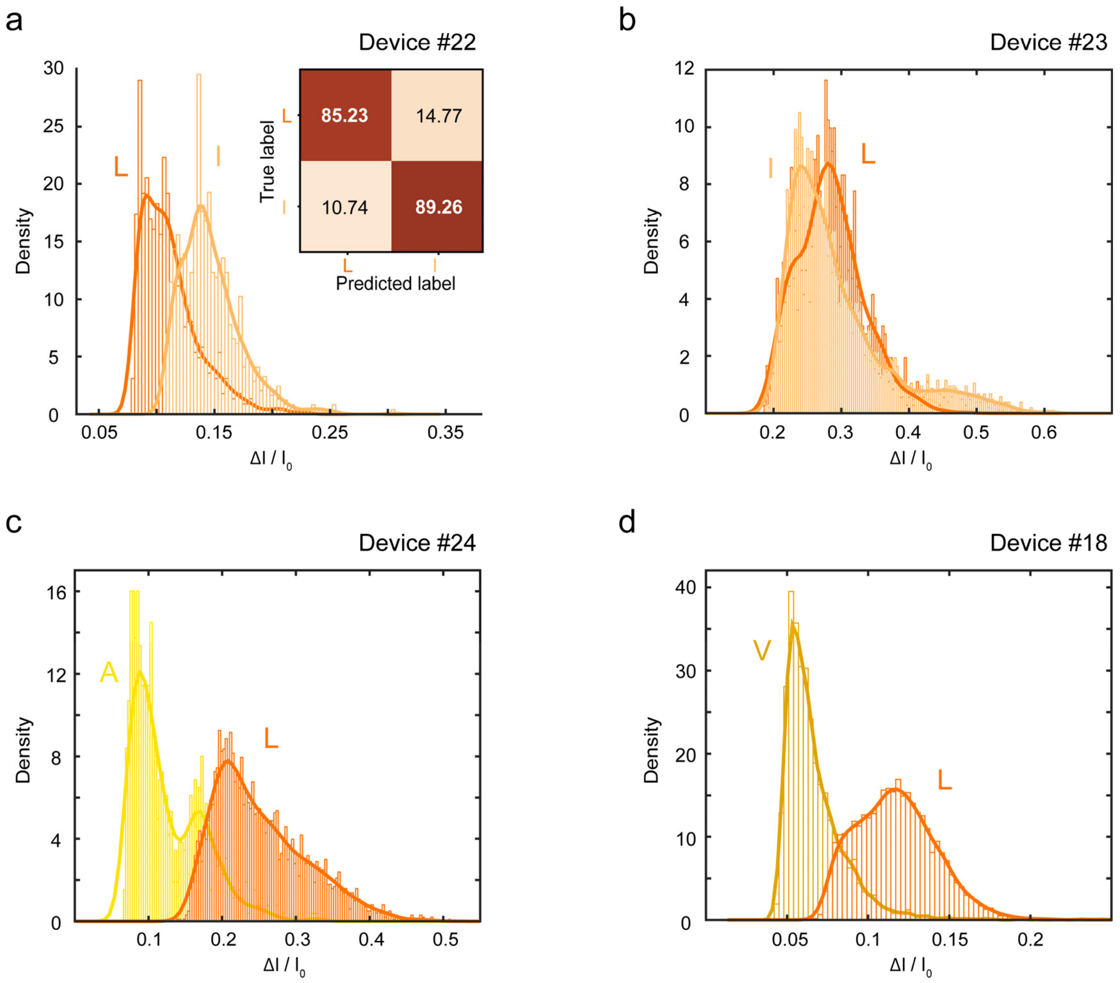

Supplementary Fig. 24. Representative reproduced experiments of the hydrophobic non-aromatic amino acids. (a-b) Reproduced experiments of L and I in Device #22 and #23. For **(a)**, the average identification accuracy is 87.25%. **(c-d)** Histograms of $\Delta I/I_0$ obtained from nanopore experiments of A and L, V and L in Device #24 and #18, respectively.

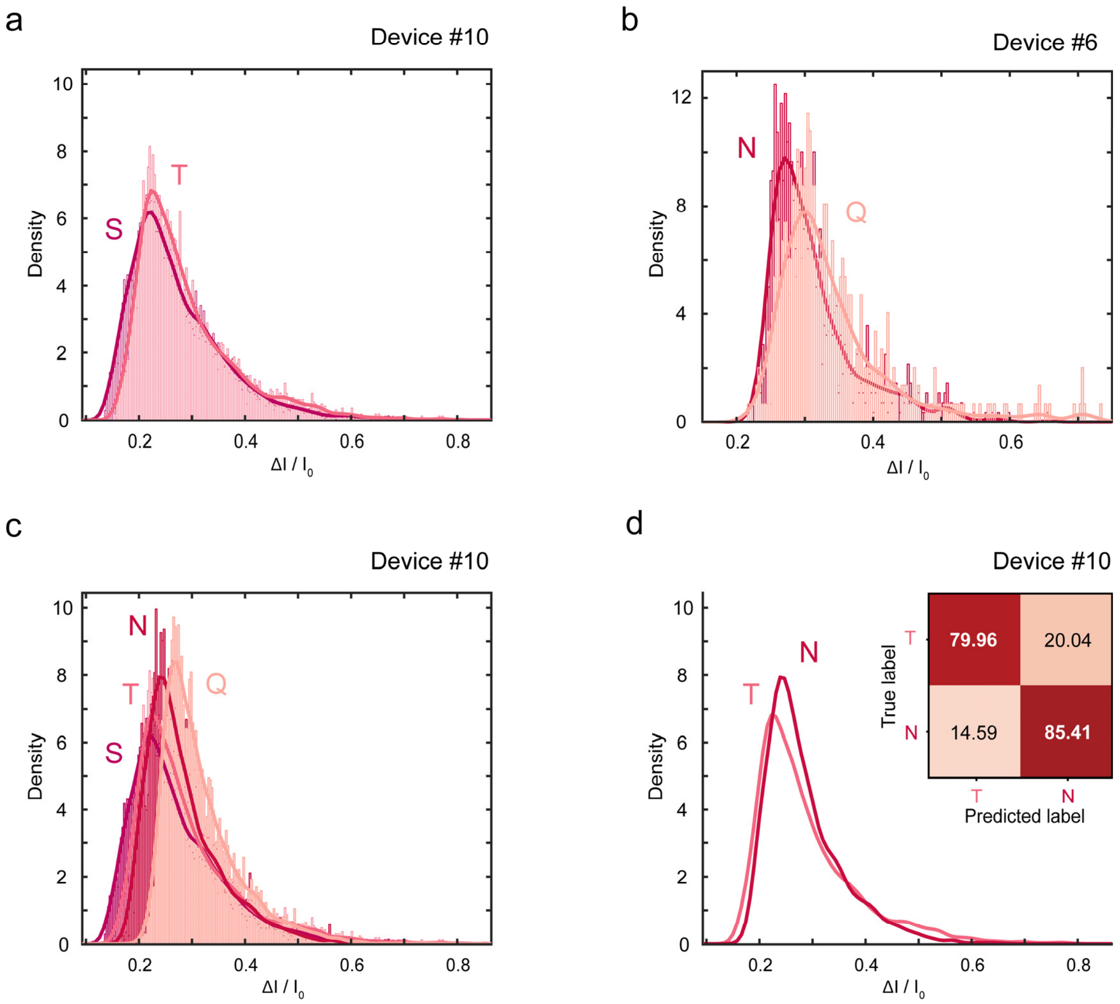

Supplementary Fig. 25. Representative reproduced experiments of polar uncharged amino acids. (a) Reproduced experiments of S and T in Device #10. **(b)** Reproduced experiments of N and Q in Device #6. **(c)** The results of S, T, N and Q in Device #10. **(d)** The results of T and N in Device #10 attached with confusion matrix, and the average identification accuracy is 82.69%.

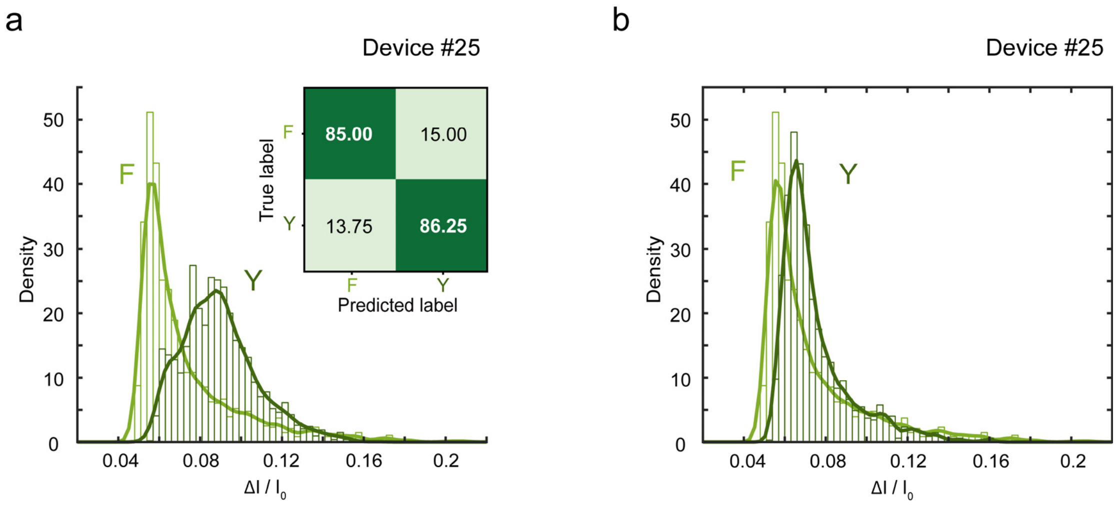

Supplementary Fig. 26. Representative reproduced experiments of hydrophobic aromatic amino acids. (a-b) The experiment of F and Y in the same device (Device #25) but with different measurement order. For **(a)**, the measurement is performed on Y first, and then on F. The average identification accuracy is 85.63%. For **(b)**, the measurement is performed on F first, and then on Y.

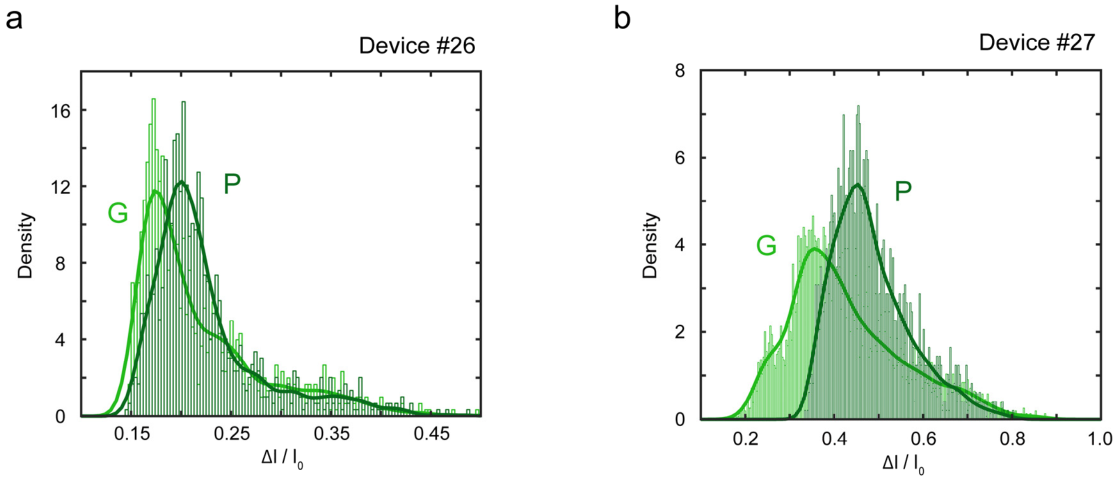

Supplementary Fig. 27. Representative reproduced experiments of G and P in Device #26 (a) and Device #27 (b).

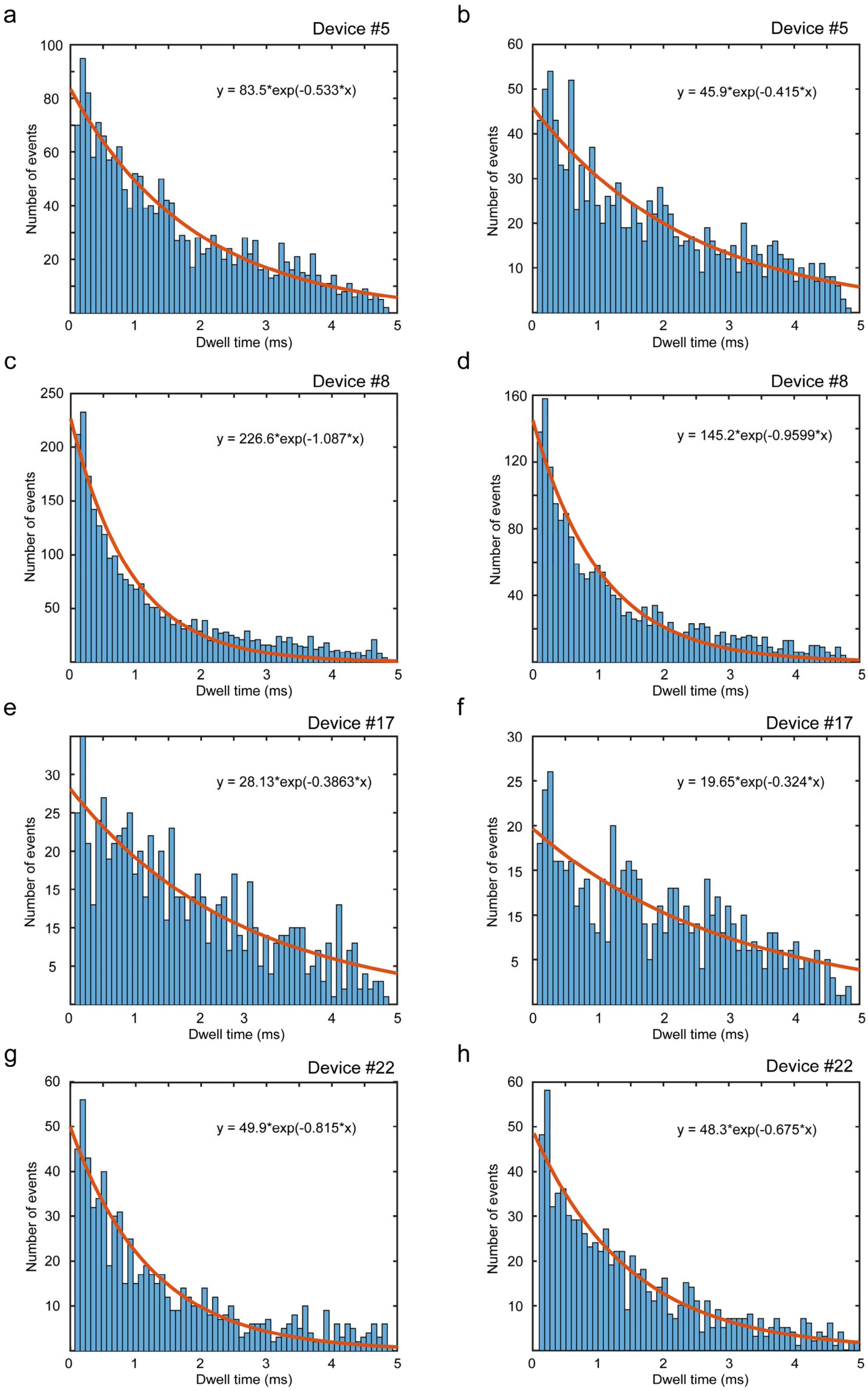

Supplementary Fig. 28. Dwell time of translocations of L and I. The data of **(a)**, **(c)**, **(e)** and **(g)** is collected for L, and the data of **(b)**, **(d)**, **(f)** and **(h)** is collected for I. All the data were fitted by the exponential decay curves. The data of **(a, b)** is from Device #5, the data of **(c, d)** is from Device #8, the data of **(e, f)** is from Device #17, the data of **(g, h)** is from Device #22.

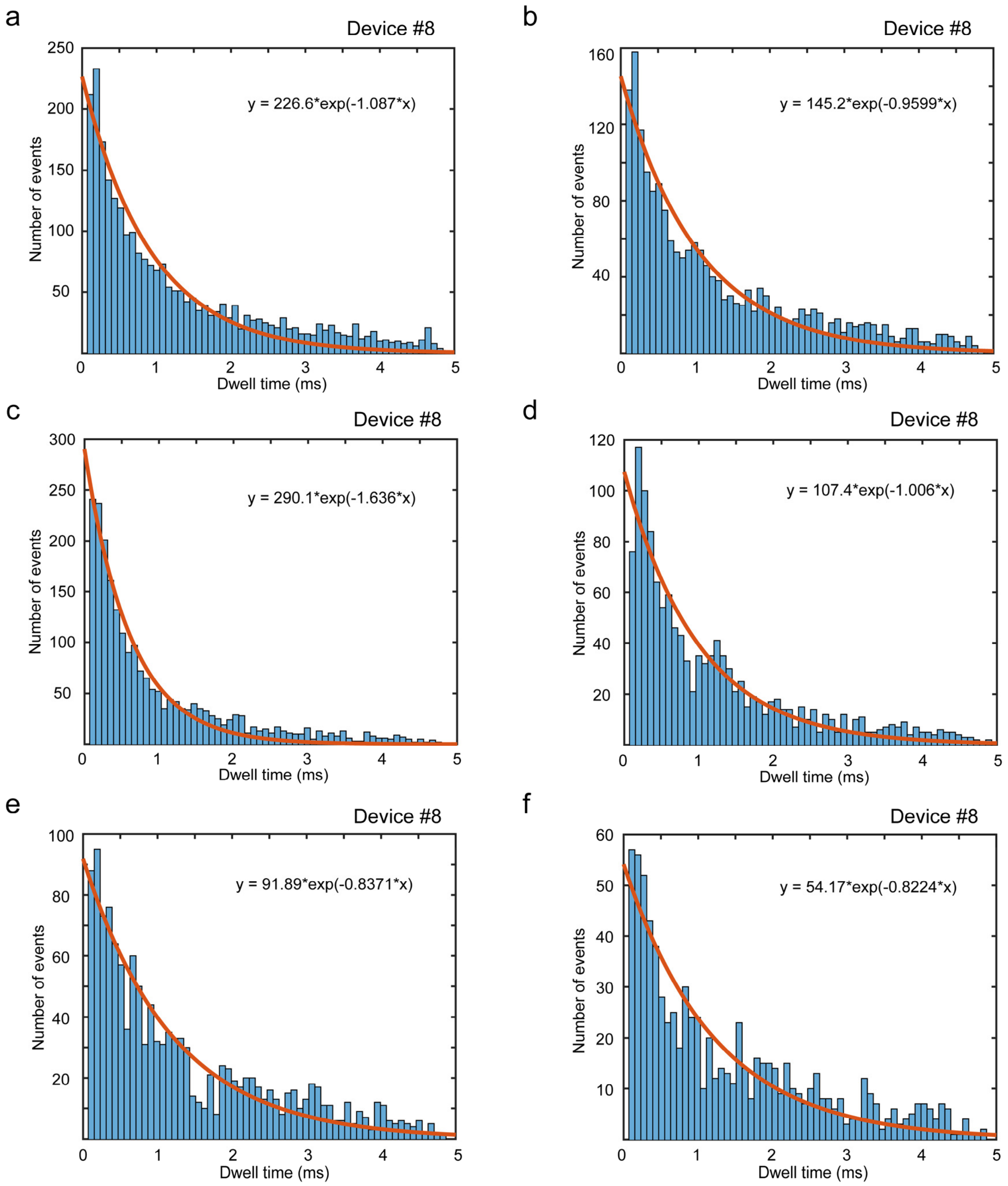

Supplementary Fig. 29. Dwell time of translocation for L, I, M, F, Y and W. The data of **(a-f)** is collected for L, I, M, F, Y and W, respectively (Device #8). All the data were fitted by exponential decay curves.

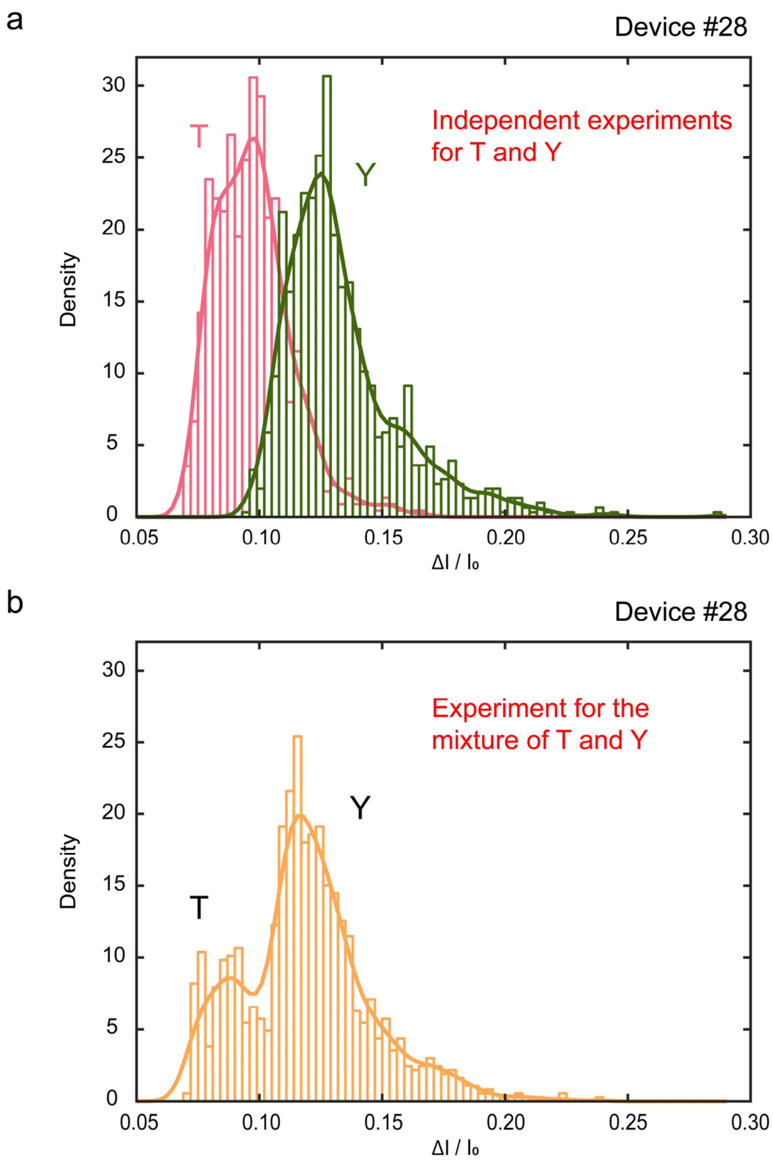

Supplementary Fig. 30. Discrimination of mixed T and Y in a MoS_2 nanopore (Device #28). Histograms of relative current blockade $\Delta I / I_0$ for individual amino acids T and Y **(a)** and the mixture solution of T and Y **(b)**. Mean peak values: for the individual T and Y, T: 0.097±0.014; Y: 0.125±0.012; for the mixture solution of T and Y, T: 0.088±0.009; Y: 0.117±0.014.

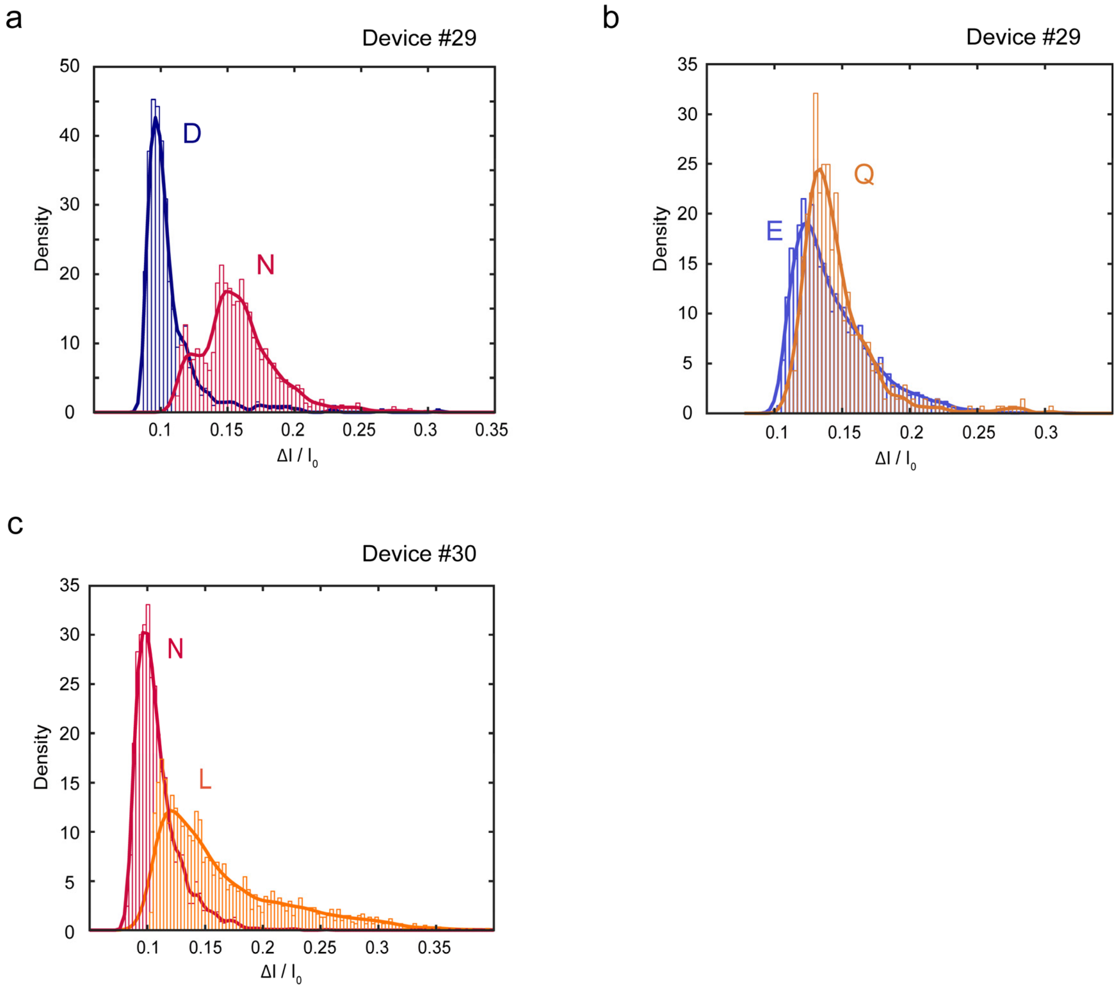

Supplementary Fig. 31. Reproduced experiments of D and N in Device #29 (a), E and Q in Device #29 (b), N and L in Device #30 (c).

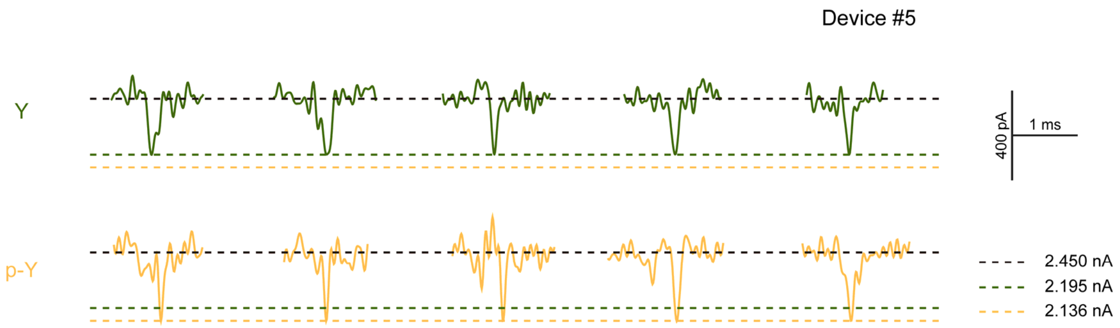

Supplementary Fig. 32. Representative fragments of current traces for the translocation of Y and p-Y (Device #5).

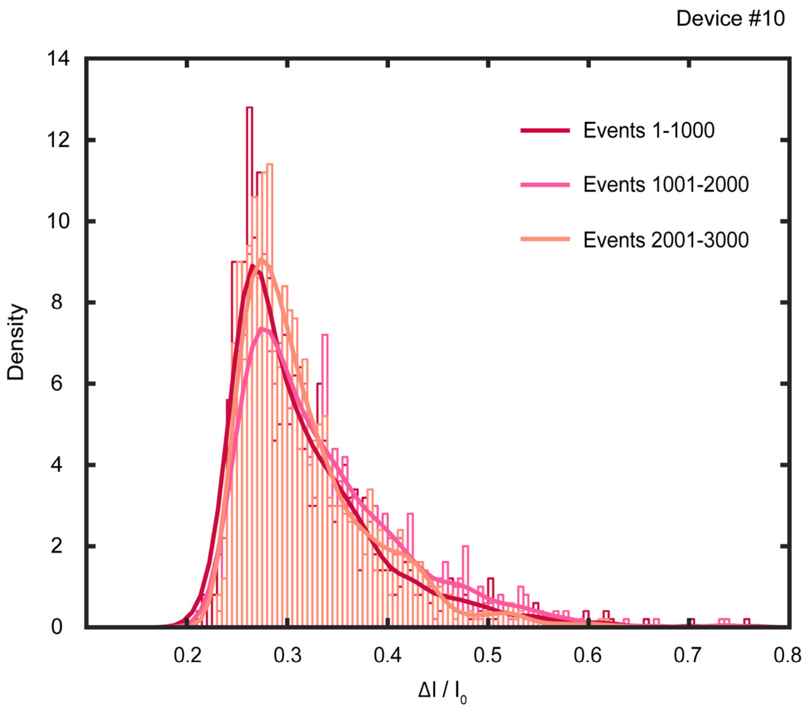

Supplementary Fig. 33. For the same amino acids (Q) in the same device (Device #10), the results of $\Delta I/I_0$ do not change over time.

Hyper-parameter	Value
Hidden units of LSTM	300
Max length of LSTM	300
Node number of FCN1	1024
Node number of FCN2	1024
Node number of FCN3	512
Node number of FCN4	Number of classes
Activation function	ReLU
Learning rate	1e-4
Batch size	32
Number of training epochs	100

Supplementary Table. 1. The hyper-parameter settings of SAAINet network.

Supplementary references

1. Feng, J. et al. Electrochemical Reaction in Single Layer MoS_2: Nanopores Opened Atom by Atom. *Nano Lett.* **15**, 3431-3438 (2015).
2. Graf, M. et al. Fabrication and practical applications of molybdenum disulfide nanopores. *Nat. Protoc.* **14**, 1130-1168 (2019).
3. Wanunu, M. et al. Rapid electronic detection of probe-specific microRNAs using thin nanopore sensors. *Nat. Nanotechnol.* **5**, 807-814 (2010).
4. Kowalczyk, S.W., Grosberg, A.Y., Rabin, Y. & Dekker, C. Modeling the conductance and DNA blockade of solid-state nanopores. *Nanotechnology* **22** (2011).
5. Feng, J. et al. Observation of ionic Coulomb blockade in nanopores. *Nat. Mater.* **15**, 850-855 (2016).
6. Cao, C. et al. Discrimination of oligonucleotides of different lengths with a wild-type aerolysin nanopore. *Nat. Nanotechnol.* **11**, 713-718 (2016).
7. Piguet, F. et al. Identification of single amino acid differences in uniformly charged homopolymeric peptides with aerolysin nanopore. *Nat. Commun.* **9**, 966 (2018).
8. Chen, H. et al. Protein Translocation through a MoS_2 Nanopore:A Molecular Dynamics Study. *J. Phys. Chem. C* **122**, 2070-2080 (2018).
9. Si, W. et al. Detergent-Assisted Braking of Peptide Translocation through a Single-Layer Molybdenum Disulfide Nanopore. *Small Methods* **4** (2020).
10. Barati Farimani, A., Heiranian, M. & Aluru, N.R. Identification of amino acids with sensitive nanoporous MoS_2: towards machine learning-based prediction. *NPJ 2D Mater. Appl.* **2**, 14 (2018).
11. Yusko, E.C. et al. Real-time shape approximation and fingerprinting of single proteins using a nanopore. *Nat. Nanotechnol.* **12**, 360-367 (2017).
12. Alonso, J.L., Cocinero, E.J., Lesarri, A., Sanz, M.E. & López, J.C. The Glycine–Water Complex. *Angew. Chem. Int. Ed.* **45**, 3471-3474 (2006).
13. Vaquero, V. et al. Alanine Water Complexes. *J. Phys. Chem. A* **118**, 2584-2590 (2014).
14. Plesa, C. & Dekker, C. Data analysis methods for solid-state nanopores. *Nanotechnology* **26**, 084003 (2015).
15. Perkins, S.J. Protein volumes and hydration effects. The calculations of partial specific volumes, neutron scattering matchpoints and 280-nm absorption coefficients for proteins and glycoproteins from amino acid sequences. *Eur. J. Biochem.* **157**, 169-180 (1986).
