# Supplementary material for: MoS2 nanopore identifies single amino acids with sub-1 Dalton resolution
Source: Nat Commun. 2023 May 20;14:2895. doi: 10.1038/s41467-023-38627-x (PMC10199900; doi:10.1038/s41467-023-38627-x)
Supplement: Supplementary file 3 — Description of Additional Supplementary Files [file 41467_2023_38627_MOESM3_ESM.pdf]

<u>Description of Additional Supplementary Files Document</u>

Supplementary Software: The Supplementary Software includes the codes of Single Amino Acids Identification Network (SAAINet), a 'README' file as guidance for running the codes and a set of data as an example.
