# Supplementary material for: MoS2 nanopore identifies single amino acids with sub-1 Dalton resolution
Source: Nat Commun. 2023 May 20;14:2895. doi: 10.1038/s41467-023-38627-x (PMC10199900; doi:10.1038/s41467-023-38627-x)
Supplement: Supplementary file 4 — Supplementary Software [file 41467_2023_38627_MOESM4_ESM.zip › Supplementary Software/README.docx]

**SAAINet**

**1. System requirements**

**Hardware Requirements:**

RAM: 32+ GB

CPU: 4+ cores, 3.3+ GHz/core

GPU: NVIDIA RTX 3070 +

**Software Requirements:**

Window10 or Ubuntu

**2. Installation guide**

cuda and cudnn

pip3 install tensorflow ==1.15

pip3 install tensorlayer ==1.11.1

**3. Demo**

**Usage**

- Data processing:

python data_processing_1.py # Read the raw data, extract event related information, and save each amino acid in a file in *amino_acid.npy* format.

python data_processing_2.py # Read the *amino_acid.npy* file, and then generate the data file (*X.npy*) and label (*Y.npy*) file required for model training

- Training:

python train_SAAINet.py # Model training, randomly select 80% for model training, and the remaining 20% for model testing, and save the test results.

- Results display:

python plot_result.py # Draw the confusion matrix according to the prediction results and real labels

**Step 1: Data Processing**

**data_processing_1.py**

**data_processing_2.py**

**Step 2: Model Training**

**train_SAAINet.py**

**Step 3: Results Display**

**plot_result.py**

**4. Instructions for use**

**Python dependencies:**

python 3.6 / 3.7

numpy 1.16.0

scikit-learn 0.19.2

matplotlib 2.2.5

tensorflow 1.15.4+nv

tensorlayer 1.11.1
